# Trained Immunity: RoadMap for drug discovery and development

**DOI:** 10.7554/eLife.108465

**Published:** 2025-12-23

**Authors:** Jelmer H van Puffelen, Callum Campbell, Irene Gander-Meisterernst, Johanna Holldack, Pauline T Lukey

**Affiliations:** 1 Kupando GmbH Schönefeld Germany; 2 Target to Treatment Consulting Ltd, Stevenage Bioscience Catalyst Stevenage United Kingdom; Murdoch Childrens Research Institute Australia; National Institute of Immunology India

**Keywords:** Trained Immunity, drug discovery, drug development, immunology

## Abstract

Trained Immunity is the nonspecific (pathogen agnostic) memory of innate immune cells, characterized by altered responses upon secondary stimulation. This review provides a RoadMap for the discovery and development of therapeutics targeting Trained Immunity, aimed at researchers with strong scientific backgrounds but limited drug development experience. The article outlines five drug development domains – epigenetic, metabolic, differentiation, inflammatory, and memory changes – that guide the identification of molecular targets, model selection, and biomarker development for the discovery and development of Trained Immunity-based therapeutics. It emphasizes the application of preclinical models and artificial intelligence in target discovery and compound screening. Additionally, the review addresses challenges in translating preclinical Trained Immunity findings to clinical trials and highlights relevant disease indications and ongoing clinical trials. This review integrates scientific findings with development strategy and thereby aims to bridge the gap between discovery and clinical application, advancing the field of Trained Immunity-based therapeutics.

## Introduction

This article serves as a focused RoadMap for discovering and developing new medicines within the field of Trained Immunity. It is of particular interest to those who are looking to translate their novel scientific discoveries into new treatments targeting Trained Immunity, rather than a systematic review. The content is specifically designed for individuals who may be unfamiliar with the translational aspects of drug discovery and development (DDD), but are experts in Trained Immunity.

The objective is to support and guide non-pharma experts in converting their scientific breakthroughs into impactful medicines that address unmet needs in diseases influenced by Trained Immunity.

## General background on Trained Immunity

**Figure 1.**
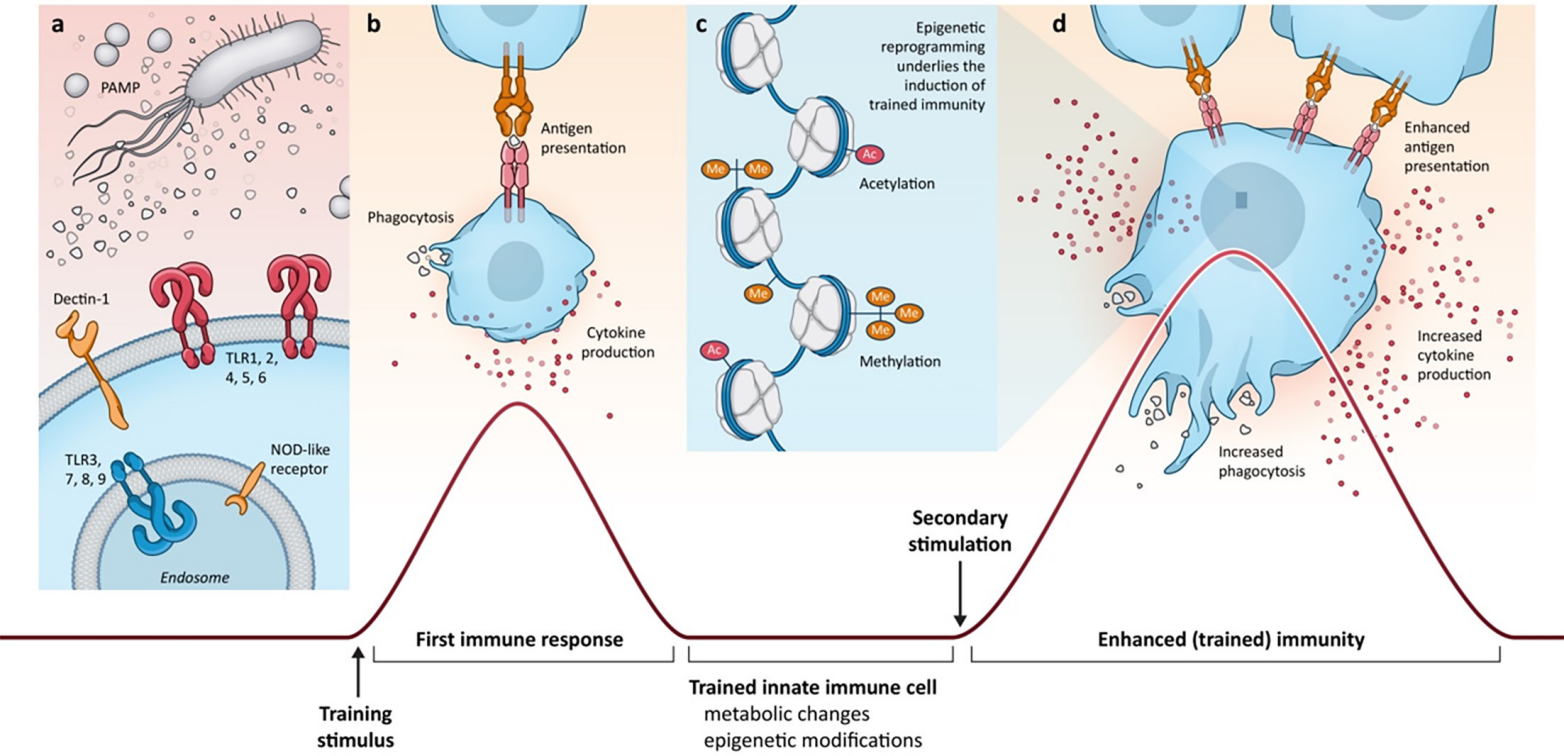
Illustration of Trained Immunity with a focus on the induction of Trained Immunity via Toll-like receptors, Dectin-1, and Nod-like receptor activation. These receptors are triggered by pathogen-associated molecular patterns (PAMP), and innate immune cells such as monocytes and macrophages are activated to produce inflammatory cytokines. Dectin-1 is the receptor that recognizes β-glucan and thereby initiates β-glucan-induced Trained Immunity. NOD-like receptors recognize Bacillus Calmette-Guérin (BCG) and thereby mediate BCG-induced Trained Immunity. Toll-like receptors recognize various types of PAMPs and can also mediate Trained Immunity (**a**). The primary immune response to infection is characterized by cytokine production and antigen presentation (**b**). The primary innate immune response dissipates, and the innate immune cell returns to baseline activation state; however, epigenetic changes, driven by metabolic reprogramming, persist at the chromatin level. This epigenetic reprogramming underlies the induction of Trained Immunity (**c**). Subsequent restimulation with an unrelated pathogen or immunological trigger initiates a Trained Immunity response that is characterized by adaptive and enhanced effector functions such as increased cytokine production, enhanced antigen presentation, and increased phagocytosis (**d**). As a secondary effect of Trained Immunity, this may lead to enhanced adaptive immune responses.

Trained Immunity is the long-lasting altered functional state of innate immune cells and is the immunologic memory of the innate immune system (Figure 1). Via Trained Immunity, innate immune cells show an altered immunologic response after a second, unrelated challenge (Netea and Joosten, 2024). Trained Immunity is independent of cells of the adaptive immune system and does not need priming or activation via antigens which are required by T cells and B cells (Ochando et al., 2023). Trained Immunity is characterized by altered responsiveness via increased production of cytokines upon secondary stimulation and the upregulation of cell surface receptors. Metabolic and epigenetic changes have an important underlying role that leads to this state of altered responsiveness. Examples of metabolic changes include an increase in glycolysis, changes in oxidative phosphorylation (OxPHOS) and glutaminolysis, altered fatty acid, cholesterol, sphingolipid, and oxylipin synthesis, and increased mitochondrial metabolism (Ferreira et al., 2024). Examples of epigenetic changes that underlie the induction of Trained Immunity are histone methylation and histone modifications which remodel the chromatin structure, leading to the change of transcriptionally repressive heterochromatin into transcriptionally permissive euchromatin (van der Heijden et al., 2018).

Trained Immunity confers nonspecific protection of the host against reinfection. This has been most extensively studied in Bacillus Calmette-Guérin (BCG) vaccination studies. The BCG vaccine was developed to protect against *Mycobacterium tuberculosis*. It has now been established that the nonspecific protective effects of the BCG vaccine against other infectious diseases are mediated (at least partly) through Trained Immunity. Other studied inducers of Trained Immunity include the cell wall component β-glucan of *Candida albicans* and oxidized low-density lipoprotein (oxLDL).

Trained Immunity is divided into central and peripheral Trained Immunity. Central Trained Immunity is the induction of Trained Immunity at the level of the bone marrow, and specifically at the level of hematopoietic stem and progenitor cells (HSPCs). These HSPCs produce innate immune cells such as monocytes. The induction of central Trained Immunity via the reprogramming of HSPCs results in long-term and systemic changes of circulating innate immune cells, with altered responsiveness (Netea and Joosten, 2024). The BCG vaccine and β-glucan induce central Trained Immunity by introducing epigenetic and metabolic changes in HSPCs (Cirovic et al., 2020; Tran et al., 2025). Furthermore, the pro-inflammatory cytokine IL-1β also induces central Trained Immunity. In fact, IL-1β plays a central role in the induction of Trained Immunity, both by BCG and β-glucan (Moorlag et al., 2020; Arts et al., 2018). Thus, the IL-1β pathway presents one of the important targets to mediate Trained Immunity responses, as will be discussed later. Peripheral Trained Immunity occurs outside of the bone marrow and the lymphoid organs. It is a tissue-specific form of Trained Immunity where innate immune cells are ‘trained’ locally and acquire altered responsiveness after a primary stimulus (Ochando et al., 2023). Training of cells residing in these peripheral tissues can also occur in non-immune cells, such as epithelial cells and fibroblasts (Knight et al., 2025).

## Trained Immunity as a therapeutic target

The immunological, metabolic, and epigenetic pathways that underlie the induction of Trained Immunity present potential therapeutic targets for drug discovery. Developing drugs that target the receptors or metabolic and epigenetic processes that mediate Trained Immunity could potentially be used in disease settings that require altered effector functions of innate immune cells, such as during immune paralysis, in cancer, or as a vaccine adjuvant. The BCG vaccine, an inducer of Trained Immunity, is already approved for the treatment of bladder cancer. Downregulating Trained Immunity responses in clinical indications with hyperinflammation, such as rheumatic diseases, may also provide novel therapeutic approaches.

**Figure 2.**
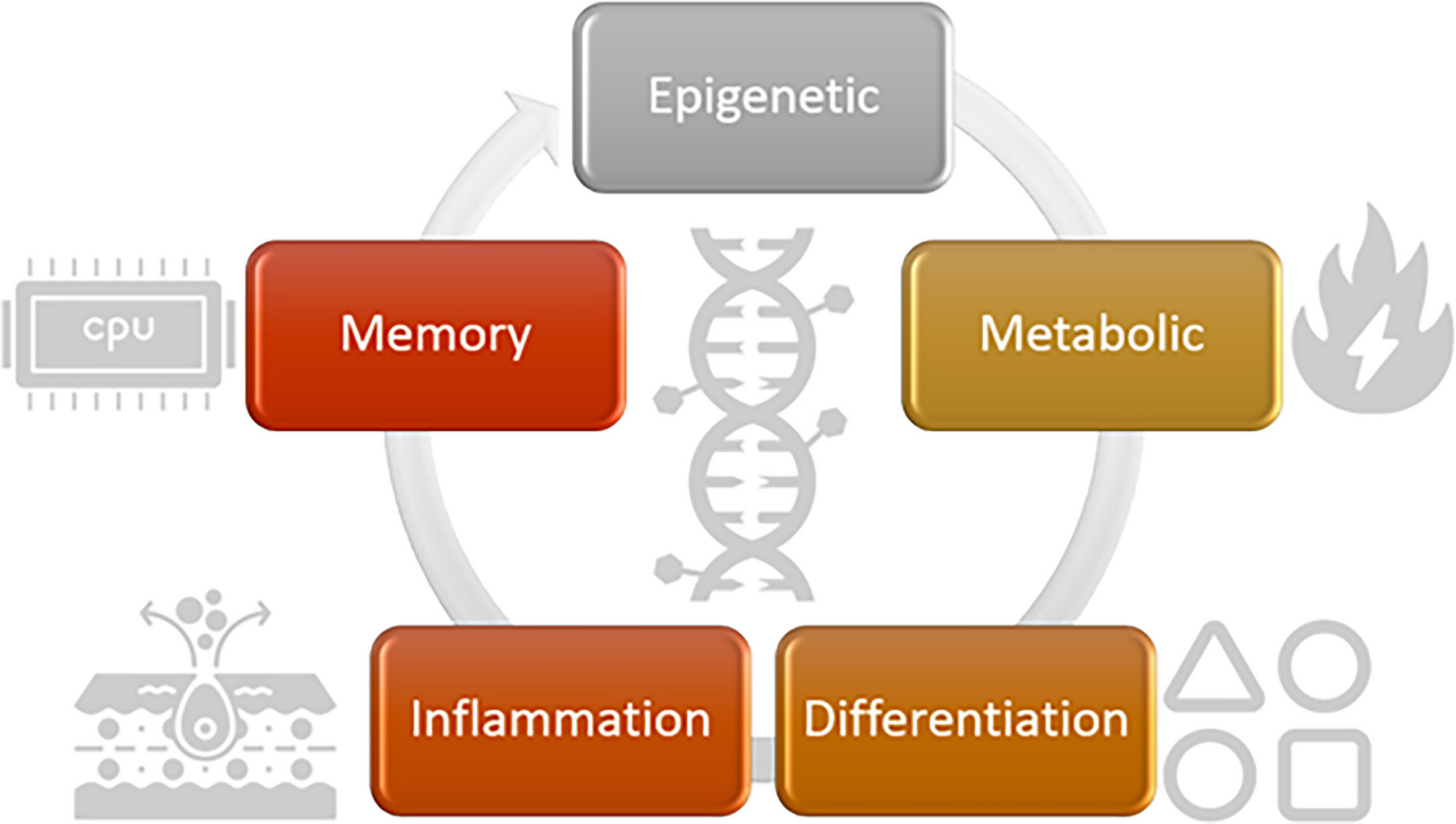
Relevant domains for Trained Immunity-targeted drug discovery and development. Five relevant domains for investigation are illustrated. Epigenetic, metabolic, and inflammatory changes, as well as differentiation and memory, are important for drug discovery and development. (Icons created by the Noun project: https://thenounproject.com/.)

For the purposes of developing a RoadMap to discovery and development of drugs targeting Trained Immunity, we use five drug development domains to facilitate discussing the different aspects of Trained Immunity (Figure 2). The drug development domains that we describe and discuss in this review are epigenetic, metabolic, differentiation, inflammatory, and memory changes that together help to describe the effects and aspects of Trained Immunity that are relevant for a RoadMap of DDD. These drug development domains can be used to:

- guide the identification and validation of molecular targets;
- design preclinical translational models (in vitro, in vivo, and in vivo);
- understand the pharmacology and dose of a candidate molecule;
- identify biomarkers of Trained Immunity.

Epigenetic changes associated with Trained Immunity are the molecular basis for the memory of innate immune cells and are embedded at the chromatin level. In addition, Trained Immunity is mediated and maintained by metabolic changes in target cells that improve their ability to react to repeated insults. Consequently, trained cells show increased phagocytic capacity, increased potential for antigen presentation (González-Pérez et al., 2025), and enhanced inflammatory responses due to increased cytokine and chemokine production upon secondary stimulation. Constitutive cytokine expression or inflammation is not typical of the trained state. In fact, following the initial priming event, the inflammatory response subsides and becomes quiescent only to react when the second event occurs. To characterize Trained Immunity responses, it is therefore necessary to study various clinical and preclinical endpoints that underpin each of these domains.

## Drug discovery and development

**Figure 3.**
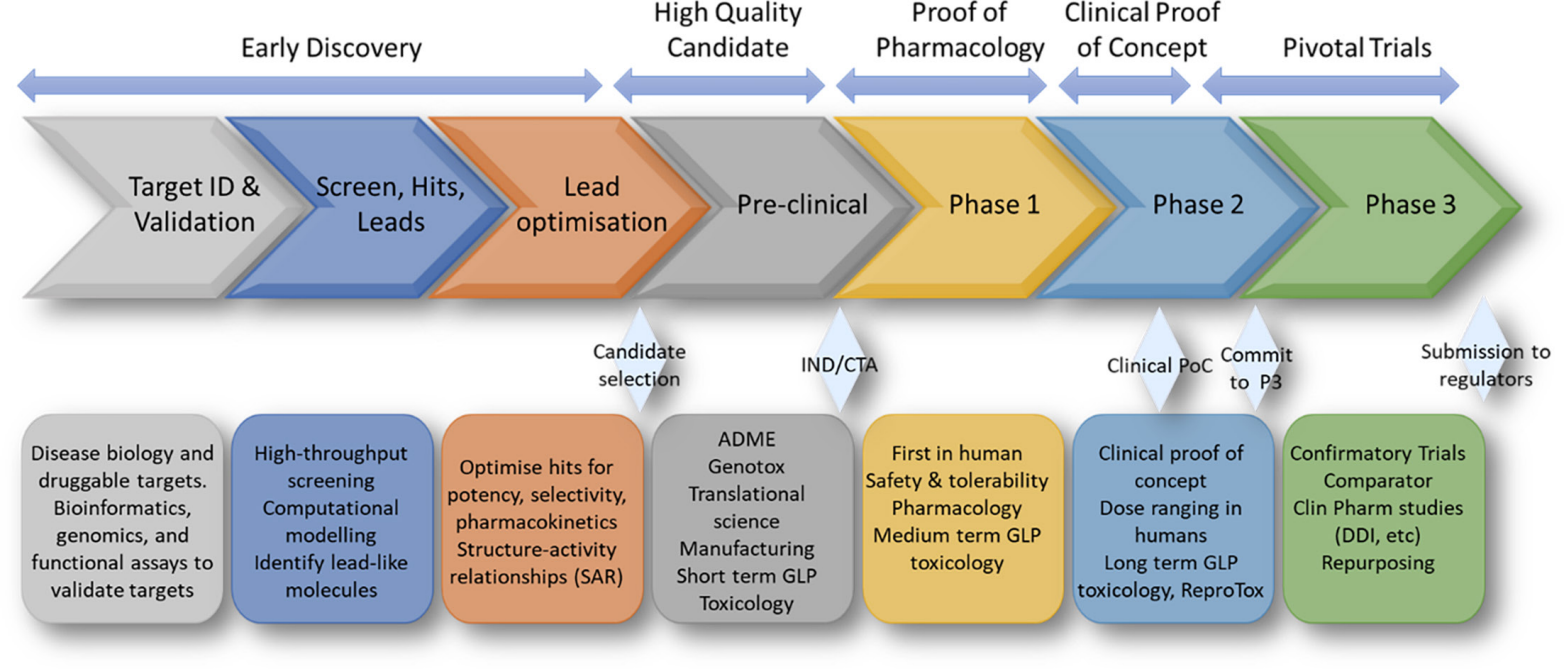
Simplified overview of a drug discovery and development pipeline. The main stages of drug discovery and development are included in the chevrons from target identification and validation through Phase 3 clinical trials. Each stage has a brief description in the boxes below the chevrons. The diamond shapes represent significant milestone achievements along the pathway. These include the selection of the single candidate that will be progressed to the clinic and the submission of the data package and clinical protocol to the regulators for approval to enter the clinic (FDA: open an IND application; EMA: CTA). Clinical proof of concept (PoC) is achieved when clinical effects of the new drug are demonstrated in patients (usually Phase 2a). The ‘commit to Phase 3’ meeting with regulators occurs when the therapeutic dose has been identified in Phase 2b and has been ratified by the regulators. At the end of Phase 3, the entirety of the data is submitted to the regulators for approval to make the new drug available to patients.

In general, DDD is a complex, multistage process that typically takes 10–15 years and costs around $2.6 billion (Figure 3).

DDD consists of several key stages that are described in detail in Appendix 1.

### DDD in Trained Immunity using AI and machine learning

**Figure 4.**
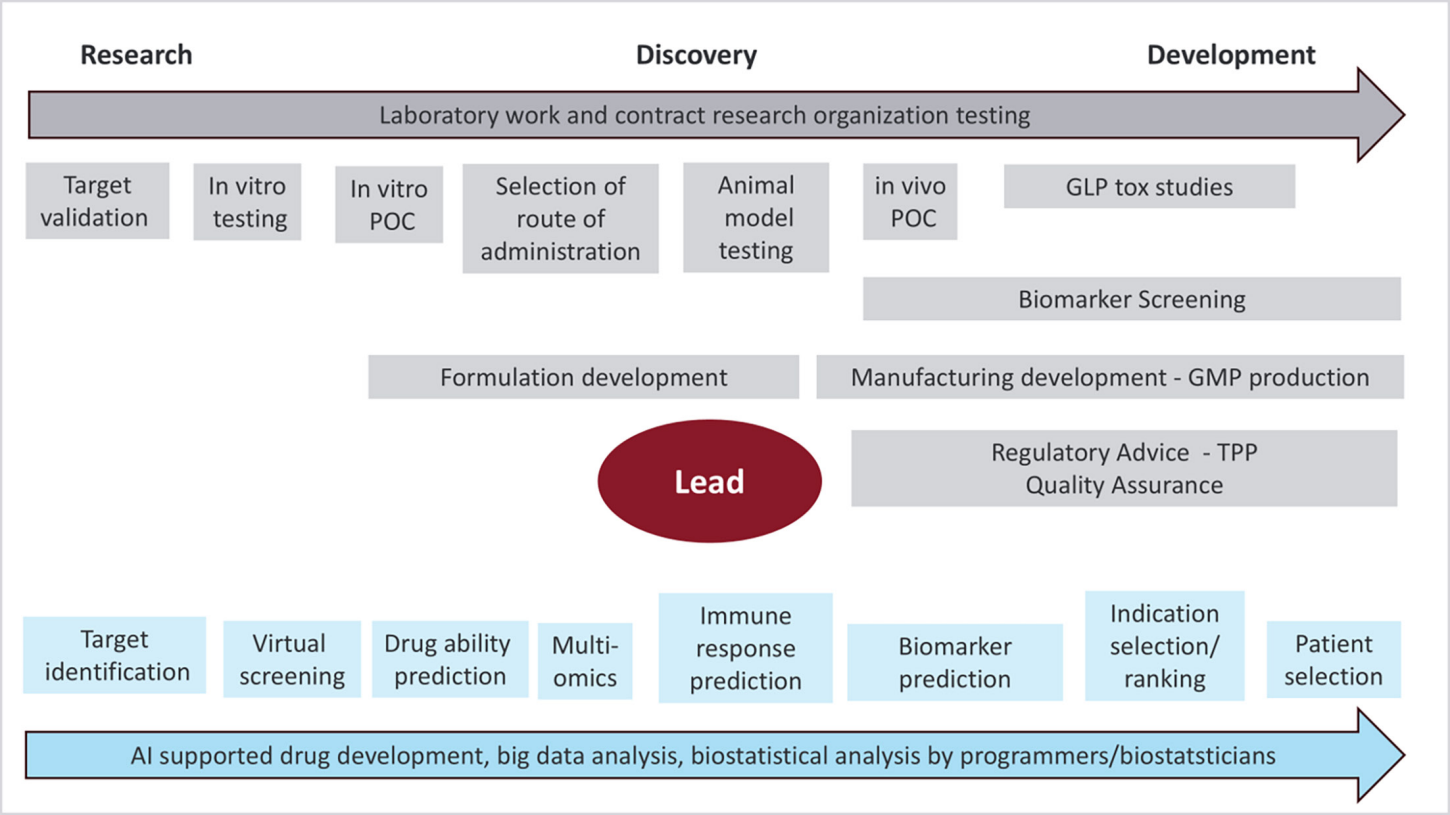
All steps from research to development up to the start of clinical development can be supported by AI. In an iterative process, data is generated in the laboratory as shown in the upper half of the figure in gray, with AI offering valuable input at each stage as shown in the lower half of the figure in blue: From target identification to the selection of nonclinical in vitro and in vivo models, and finally, the identification of patient subsets and biomarker and outcome assessment once a drug candidate progresses to the clinical phase. Once the lead has been identified, development incorporates stringent adherence to development standards such as the GLP, GMP, and GCP guidelines and standards. Regulatory advice needs to be included to streamline drug development and entry into Phase 1 and to start building a target product profile. Drug discovery and development in Trained Immunity seeks to leverage and modify the innate immune system to achieve a durable and balanced immune response.

The immune system is a complex biological system which poses a challenge to identifying lead candidates for drug development, and AI/ML could play a key role in advancing discoveries in this field. Notably, the FDA acknowledges the importance of AI in the drug discovery process in a recent extensive discussion paper (FDA, 2025). Figure 4 illustrates the steps involved from research and discovery to drug development, highlighting the potential AI-supported aspects in the lower half.

Drugs that enhance Trained Immunity may be developed to protect against infections, assist the immune system in combating cancer, and prevent metastasis (Ding et al., 2023; Vuscan et al., 2025). On the other hand, drugs that regulate Trained Immunity may be developed for diseases involving hyperinflammation such as autoimmune or autoinflammatory diseases (Mora et al., 2023; Badii et al., 2022).

#### Target identification

Machine learning models may be used to pinpoint proteins or pathways implicated in immune memory formation, accelerating the identification of therapeutic targets for Trained Immunity. Additionally, AI can inform at which level of Trained Immunity (i.e. central or peripheral Trained Immunity; Netea et al., 2020a), the target has the most potential to reach its therapeutic effect.

AI may be used to screen large databases of molecular and genetic data to identify novel inducers of Trained Immunity without triggering excessive inflammation. For example, machine learning algorithms have been used to discover the role of Toll-like receptor (TLR) agonists and other immune-modulating molecules, such as nanoparticle-based adjuvants in Trained Immunity (Abiri et al., 2021; Zhang et al., 2024; Sobhani et al., 2024; Knight et al., 2024).

AI may be used to simulate immune system dynamics to identify molecules that induce Trained Immunity through epigenetic and metabolic reprogramming. This includes screening compounds that target pathways like mTOR or NLRP3 inflammasome activation (Ferreira et al., 2022a).

#### Hit identification

Machine learning algorithms can be used to prioritize hit compounds based on their potential to induce or regulate Trained Immunity phenotypes in vitro and in vivo. This approach can help pinpoint noninflammatory inducers of Trained Immunity, e.g., glucocorticoids (Knight et al., 2024) could be particularly valuable for therapeutic applications. For example: A-HIOT (Automated Hit Identification and Optimization Tool) integrated chemical and protein space-driven stacked ensemble models to refine hit selection for CXC chemokine receptor 4. It provided a reliable approach for bridging the gap between ligand-based and structure-based virtual screening in finding the optimized hits for CXCR4 (Kumar and Acharya, 2022).

#### Multi-omics

AI algorithms may help to integrate and analyze various omics datasets (genomics, epigenomics, transcriptomics, and metabolomics) to identify the most promising indications for Trained Immunity-based interventions. This comprehensive approach can reveal complex interactions and mechanisms underlying Trained Immunity in different disease contexts. SIMON AI software accelerates the discovery of human immune memory responses to viruses by integrating multi-omics analysis. While it focuses on viral immunity, this approach could be applicable to Trained Immunity as viral stimuli can also induce Trained Immunity and antiviral immune responses such as increased production of type 1 interferons can be augmented in Trained Immunity settings (Taks et al., 2022; Tomic et al., 2022; Koeken et al., 2021).

#### Immune response and Trained Immunity prediction

AI models, like the AI-Cell tool, can predict how human immune cells might respond to nanomedicines, which is crucial for developing safer and more personalized gene therapies that could induce Trained Immunity. These predictive models can help anticipate potential immune reactions to treatments, allowing for optimization of therapies that harness Trained Immunity (Chandler et al., 2022; Priem et al., 2020).

Machine learning frameworks like scifAI may be used to analyze millions of images of interacting immune cells to measure changes caused by specific antibodies or compounds (Shetab Boushehri et al., 2023). This approach can predict T cell activation and cytokine production, which are key aspects of Trained Immunity responses. Universal LIPSTIC (uLIPSTIC) offers a way to address the challenge of investigating transient interactions between cells of the immune system that come into close physical proximity (Nakandakari-Higa et al., 2024).

Machine learning models specifically applied to multi-omics data can also be used to classify patients into Trained Immunity responders and nonresponders, as was shown by Moorlag et al., 2024. In this study, the researchers generated a model that could classify patients into Trained Immunity responders and nonresponders, and the model with the most predictive value included chromatin accessibility profiles at baseline (before BCG vaccination), host factors, and vaccination day.

## Molecular targets and their challenges

Potential targets in Trained Immunity currently under investigation include but are not limited to:

- Epigenetic regulators: Modulating histone modifications, DNA methylation, and histone lactylation can influence Trained Immunity responses.
- Metabolic pathways: Enzymes involved in glycolysis, OxPHOS, glutaminolysis, autophagy, fatty acid metabolism, and lipid metabolism play a role in immune cell reprogramming.
- Pattern recognition receptors (PRRs): These extracellular or intracellular receptors detect pathogen-associated molecular patterns and can be targeted to regulate Trained Immunity.
- Cytokine signaling pathways: Controlling inflammatory cytokines like IL-1β and IL-4 may help fine-tune Trained Immunity, e.g., targeting IL-1β signaling via the IL-1 receptor (IL-1R).

To date, several molecular targets and mechanisms that regulate Trained Immunity have been identified. These targets, pathways, and mechanisms can be targeted by numerous Trained Immunity-regulating compounds (Table 1).

**Table 1.** Summary of the main classes of drug targets that target Trained Immunity and the main drug development domains they modulate.

Target class	Main affected drug development domain
**Pattern recognition receptors** Toll-like receptors (TLRs) (Alexopoulou and Irla, 2025) NOD-like receptors (NLRs) (Kleinnijenhuis et al., 2012) C-type lectin receptors (CLRs) (Moorlag et al., 2020; Moerings et al., 2021)	Epigenetic Metabolic Differentiation Inflammatory Memory
**Cytokines and cytokine receptors** IL-1β and IL-1R (Moorlag et al., 2020; Teufel et al., 2022) IL-4 and IL-4R (Schrijver et al., 2023)	Metabolic Inflammation Differentiation
**Epigenetic enzymes** Histone acetyltransferases (Ziogas et al., 2025; Fanucchi et al., 2021), Histone deacetylases (Cheng et al., 2014; Mourits et al., 2021a), Histone methyltransferases (Keating et al., 2020b; Mourits et al., 2021b) Histone demethylases (Arts et al., 2016) Lactyltransferase (p300) (Ziogas et al., 2025)	Metabolic Epigenetic Memory
**Metabolism** Hexokinase (Cheng et al., 2014) Succinate dehydrogenase (Domínguez-Andrés et al., 2019) Acetyl-CoA carboxylase (Arts et al., 2016) Glutaminase (Scarpa et al., 2025)	Metabolic

PRRs can be stimulated or inhibited to regulate Trained Immunity responses (Alexopoulou and Irla, 2025; Owen et al., 2020). These PRRs include TLRs, NOD-like receptors (NLRs), and C-type lectin receptors (CLRs) (Ochando et al., 2023; Kleinnijenhuis et al., 2012; Moerings et al., 2022). PRRs may be intracellular or extracellular. Examples of intracellular receptors that mediate Trained Immunity responses are NOD1, NOD2, TLR3, TLR7, and TLR9. Examples of extracellular receptors are Dectin-1, TLR2, TLR4, and TLR5.

In addition to PRRs, receptors that can be bound by cytokines or growth factors can also mediate Trained Immunity responses. For example, IL-1β induces Trained Immunity via the IL-1R, and IL-4 mediates Trained Immunity via the IL-4 receptor (both type 1 and type 2 IL-4 receptors). IL-1β plays an important role in mediating central Trained Immunity (Moorlag et al., 2020; Teufel et al., 2022; Flores-Gomez et al., 2024; Mitroulis et al., 2021); therefore, it represents an important target in disease indications where Trained Immunity contributes to immunopathology, such as in autoimmunity and transplantation (Ochando et al., 2023). IL-1R can be targeted with a recombinant interleukin 1 receptor antagonist protein which is already approved and on the market (Anakinra). Furthermore, the IL-1β protein itself can be inhibited using canakinumab, which is approved for several auto-inflammatory diseases by the FDA and EMA.

The enzymes that regulate epigenetic changes that modulate Trained Immunity responses are typically located in the nucleoplasm. Thus, to target epigenetic enzymes such as histone acetyltransferases, histone deacetylases, histone methyltransferases, or histone demethylases, the active pharmaceutical ingredient should be able to reach the nucleoplasm. This poses a challenge for drug development. Small molecules that are lipophilic are suitable because they can reach the nucleoplasm via passive diffusion through the nuclear envelope. Moreover, some biological drugs with a molecular weight under 40 kDa can pass through nuclear pore complexes to reach the nucleoplasm (Klughammer et al., 2024). Thus, the lipophilic characteristic and the size of the small molecule are important aspects that allow diffusion to the nucleosome. The G9a histone methyltransferase modulates Trained Immunity responses via methylation of histone 3 lysine 9 (H3K9) and can be inhibited by BIX-01294 leading to enhanced Trained Immunity responses in human monocytes (Mourits et al., 2021b). Its small size (approximately 400 Da) and lipophilicity allow it to cross both the plasma membrane and the nuclear envelope efficiently. Sirtuin 1 is another example of an epigenetic enzyme that regulates the induction of Trained Immunity (Mourits et al., 2021a). This enzyme is a histone deacetylase. Activation of Sirtuin 1 using resveratrol (a natural polyphenol) enhanced the Trained Immunity effect induced by BCG (Bulut et al., 2025).

Metabolic regulation of Trained Immunity also occurs intracellularly in the cytoplasm of target cells. Targeting metabolic processes and enzymes therefore also requires a pharmaceutical active ingredient that can reach the cytoplasm. Some potential metabolic targets that are involved in Trained Immunity include (Ferreira et al., 2024; Ferreira et al., 2022a; Ziogas et al., 2025; Arts et al., 2016; Domínguez-Andrés et al., 2019; Hao et al., 2025; Bekkering et al., 2018):

- **Glycolysis**: The enzyme **hexokinase** is a critical regulator of glucose metabolism and has been implicated in Trained Immunity responses (Cheng et al., 2014; Keating et al., 2020a).
- **Tricarboxylic acid (TCA) cycle: Succinate dehydrogenase** influences immune cell activation and epigenetic modifications (Domínguez-Andrés et al., 2019).
- **Lipid metabolism: Acetyl-CoA carboxylase** plays a role in fatty acid synthesis, which affects immune cell function (Arts et al., 2016).
- **Glutaminolysis: Glutaminase** regulates glutamine metabolism, which is essential for Trained Immunity (Scarpa et al., 2025).

Furthermore, at the level of gene expression, there are several transcription factors that regulate Trained Immunity responses. Notable transcription factors that regulate Trained Immunity are HIF1α, NF-κB, and RORα (Kilic et al., 2024; Mulder et al., 2019; Hu et al., 2022). Some of these transcription factors are also involved in regulating the metabolic changes that occur in Trained Immunity (Cheng et al., 2014).

Some examples of compounds that regulate Trained Immunity are shown in Table 2.

**Table 2.** Trained Immunity-regulating compounds.

Description of Trained Immunity regulating compound	Type	Trained Immunity target	Inducing or inhibiting Trained Immunity*	Cellular location of Trained Immunity target	Status	Reference
**BCG vaccine**	Live-attenuated vaccine	NOD2 receptor, TLR2, TLR4	Inducing	Intracellular	Marketed	Moorlag et al., 2024
**MV130**	Whole heat-inactivated bacteria (90% Gram-positive, 10% Gram-negative)	Combination of TLRs and NLRs	Inducing	Intracellular and extracellular	In development	Brandi et al., 2022
**MDP combined with HPV E7 peptide encapsulated by polylactic-co-glycolic acid PLGA nanoparticles**	Nanoparticle	NOD2 receptor	Inducing	Intracellular	Experimental	Li et al., 2023
**MTP**_**10**_ **-HDL**	Nanoparticle	NOD2 receptor	Inducing	Intracellular	In development	Priem et al., 2020
**PEG-PDLLA polymersome containing β-glucan**	Polymersome	Dectin-1	Inducing	Extracellular/transmembrane receptor	In development	Wauters et al., 2024
**PLGA nanoparticles containing β-glucan**	Nanoparticle	Dectin-1	Inducing	Extracellular/transmembrane receptor	Experimental	Ajit et al., 2022
***Saccharomyces cerevisiae*** **-derived whole glucan particles containing β-glucan**	Sonicated yeast particles	Dectin-1	Inducing	Extracellular/transmembrane receptor	Experimental	Horneck Johnston et al., 2024
**BIX‐01294**	Small molecule	G9a histone methyltransferase	Inducing	Nucleoplasm	Experimental	Mourits et al., 2021b
**Monophosphoryl lipid A (MPLA)**	Modified lipid	TLR4	Inducing	Extracellular/transmembrane receptor	Experimental	Owen et al., 2022
**Oxidized low-density lipoprotein (OxLDL)**	Lipoprotein	TLR4	Inducing	Extracellular/transmembrane receptor	Experimental	Bekkering et al., 2014
**CpG-ODN**	Oligonucleotide	TLR9	Inducing	Intracellular	Experimental	Owen et al., 2022
**β-Glucan in arabinoxylan**	Hemicellulose	Complement receptor 3 (CR3)	Inducing	Extracellular/transmembrane	Experimental	Moerings et al., 2022; Patin et al., 2019
**Fusion protein of apolipoprotein A1 and IL4**	Lipid nanoparticle	IL-4 receptor (type 1 and type 2)	Inducing	Extracellular/transmembrane	In development	Schrijver et al., 2023
**Anakinra**	Recombinant protein	IL-1 receptor	Inhibiting	Extracellular	Marketed	Flores-Gomez et al., 2024; Mitroulis et al., 2018; Ciarlo et al., 2020
**Canakinumab**	Monoclonal antibody	IL-1β protein inhibitor	Inhibiting	Extracellular	Marketed	Xu et al., 2025
**5′-Deoxy-5′methylthio adenosine (MTA)**	Synthetic organic compound	Histone methyltransferase inhibitor	Inhibiting	Nucleoplasm	Experimental	Quintin et al., 2012
**Resveratrol**	Natural polyphenol	Sirtuin 1 (histone deacetylase) activator	Inducing	Nucleoplasm	Marketed (as supplement)	Mourits et al., 2021a; Bulut et al., 2025; Wang et al., 2021a

* The aim of inducing or inhibiting Trained Immunity is to change the inflammatory state in a certain (disease) setting. Depending on the clinical indication, modulation of Trained Immunity responses can either boost or diminish inflammatory responses. For example, boosting immune responses via Trained Immunity can change the equilibrium during immunoparalysis. On the contrary, in a chronic hyperinflammatory state, inhibiting Trained Immunity responses could potentially serve as a tool to change the equilibrium the other way around.

## Formulation

The pharmaceutical formulation facilitates the biodistribution, clearance, and pharmacological effect of the Trained Immunity-regulating drug. The formulation also plays a key role in the delivery of the drug to the target organ, target receptor, or target protein. As discussed previously, the receptors, proteins, and enzymes that regulate Trained Immunity processes can be located in the nucleoplasm, intracellular, or as an extracellular or transmembrane receptor. Moreover, depending on the clinical indication, the target cell or organ will determine the required characteristics of the pharmaceutical formulation as well. For example, a Trained Immunity inducer that is to reach the HSPCs in the bone marrow will likely need to be administered as an intravenous drug, whereas a mucosal vaccine, targeting immune cells in mucosa, does not have to be administered intravenously per se, but can be applied topically in the mucosa. Thus, depending on the disease context, target tissue, and the distinction between central versus peripheral Trained Immunity, the delivery system of the Trained Immunity-regulating compound will be different. The administration, location, and delivery vehicle should be designed to ensure that the target tissue or cell type is targeted with minimal side effects. In case a long-term effect on Trained Immunity is required, delivery to the bone marrow, as well as stability in blood and bone marrow tissue, is important. Such a stable formulation prevents the degradation of the compound during administration and preserves its biological effect. It is therefore important to assess the stability of the Trained Immunity-regulating compound in different matrices such as plasma, whole blood, and liver microsomes and to perform early absorption, distribution, metabolism, and excretion (ADME) studies.

Early nonclinical work is often done with simple formulations, e.g., compounds dissolved in DMSO. However, the formulation may have an impact on the pharmacology and kinetics of an active substance. Depending on the route of administration, nanoparticle-based systems offer versatility in payload incorporation, cellular targeting, and ability to modulate immune responses, making them particularly well suited for inducing and regulating Trained Immunity (van Leent et al., 2022).

### Nanoparticles and polymerosomes

To achieve the therapeutic potential of a drug that regulates Trained Immunity, it must be capable of reaching organs and tissues that contain reservoirs of innate immune cells, such as the bone marrow, spleen, and lymphoid organs. Preclinical biodistribution studies can be performed to assess the biodistribution pattern of drugs. Currently, the biodistribution patterns of most Trained Immunity-regulating drugs have not been fully elucidated. Two studies have used labeling of nanoparticles and polymerosomes (a bilayer vesicular system with a central aqueous core that can carry compounds such as drugs, enzymes, or nucleic acids) with the radioisotope zirconium-89 (^89^Zr) to track the in vivo distribution of Trained Immunity-regulating drugs in mice and nonhuman primates. Specifically, Scheerstra et al. performed an in vivo bioavailability study, in which polymerosome-containing β-glucan was radiolabeled. They administered the radiolabeled polymerosomes intravenously and subsequently tracked their distribution in mice and nonhuman primates using PET imaging techniques. They showed that the polymerosomes were accumulating in the spleen, bone marrow, and liver and were taken up by monocytes and macrophages (Wauters et al., 2024). Furthermore, Priem et al. also used ^89^Zr labeling to perform in vivo biodistribution studies to assess the distribution of muramyl tripeptide-high-density-lipoprotein (MTP-HDL) in mice and nonhuman primates (Priem et al., 2020). They showed that the MTP-HDL nanoparticle accumulated in the bone marrow and spleen, and they were also able to show liver uptake. Therefore, nanoparticles and polymerosomes appear to be efficient formulations to modulate central Trained Immunity. It should be noted that the spleen might have a limited role in the establishment of central Trained Immunity responses, as the removal of the spleen of mice did not influence the proinflammatory cytokine production of peritoneal cells in an in vivo β-glucan-induced Trained Immunity mouse model (Ferreira et al., 2022b).

### The BCG vaccine

Several formulation types of Trained Immunity-regulating agents are in preclinical development, and only a few are approved for human use. One example of a Trained Immunity-inducing therapy in use in humans is the BCG vaccine, a live-attenuated vaccine originally designed to protect against *M. tuberculosis* infection. Numerous in vivo and ex vivo studies have shown that the BCG vaccine induces Trained Immunity in humans (Moorlag et al., 2024; Kong et al., 2021). BCG vaccination protects against other infectious diseases mediated via Trained Immunity responses. The BCG vaccine is also used as an adjuvant immunotherapy to treat patients with non-muscle invasive bladder cancer after the bladder tumor has been removed during surgery. As a bladder cancer treatment, the BCG vaccine is administered as an intravesical administration, which is a topical administration on the bladder urothelium (Singh et al., 2021). Intravenous administration of BCG is unacceptable due to the safety risks of administering a live mycobacterium in the circulation.

It should be noted, however, that live (attenuated) Trained Immunity inducers have been reported to produce a stronger Trained Immunity response compared to inactivated (heat-killed or gamma-irradiated) Trained Immunity inducers (Sánchez-Morales et al., 2025; Arts et al., 2015). Importantly, BCG administered in the bladder was shown to colonize the bone marrow, both in mice and in humans. This colonization then leads to epigenetic reprogramming of HSPCs and Trained Immunity induction (Daman et al., 2025). In this sense, the delivery and the stability of a live Trained Immunity inducer appears to be beneficial for inducing central Trained Immunity. It can be hypothesized that non-live Trained Immunity inducers have a less persistent effect on the induction of Trained Immunity in the bone marrow.

### Temporal control of Trained Immunity using controlled release nanoparticles

A new approach is the development of controlled-release nanoparticles for a long-term, sustained induction of Trained Immunity. This approach uses polylactic-co-glycolic acid (PLGA) nanoparticles that encapsulate β-glucan as a Trained Immunity inducer. In an in vitro assay at a pH of 7.4, it was shown that the nanoparticles showed a sustained release profile of β-glucan over a period of up to 4 weeks (Ajit et al., 2022). The advantage of using such a long-term sustained release approach is the establishment of a more durable Trained Immunity phenotype which has the potential to increase protection against infectious diseases for a longer period. Inducing Trained Immunity using such a sustained release approach may be most beneficial at the start of the flu season, in particular.

### Potential immunological side effects caused by excipients

It is important to note that certain excipients of the formulation may also induce unwanted immunological side effects (Saxena et al., 2025). This is especially the case for nanoparticle-based approaches that use PEGylation to increase the pharmacokinetic and pharmacodynamic profile of the drug. PEGylation is the process of attaching polyethylene glycol (PEG) to a drug, to improve their half-life and circulation time (Harris and Chess, 2003). Screening for immunological side effects may be advisable in the drug development process, in particular for novel drugs that target Trained Immunity.

## Models and biomarkers

The use of models and biomarkers in the context of DDD changes as the asset proceeds along the pipeline. Initially, the focus of these models is on target identification and target validation. However, after candidate selection, the focus of the models becomes translational science. This means that the objectives of the models change compared to the target validation phase. The objective of translational science is to understand the pharmacology of the molecule under development, rather than understanding the basic mechanisms of Trained Immunity. The focus becomes the molecule rather than the target of Trained Immunity. This change in focus is illustrated in this section of the review.

The design of translational science models and the use of translational biomarkers depend on knowledge of the basic mechanisms of Trained Immunity and the molecular target of the candidate molecule. The question to be answered therefore changes from ‘what is the role of the molecular target in Trained Immunity?’ to ‘what is the effect of the candidate molecule on Trained Immunity?’. Translational science models are therefore designed to fully display the characteristic effects of the candidate molecule on Trained Immunity.

The cellular components of Trained Immunity include monocytes, macrophages, natural killer cells, and granulocytes. In addition, the research domains that are relevant for DDD in Trained Immunity include epigenetic, metabolic, and inflammatory changes. Therefore, in designing relevant pharmacological models, the cell types involved and potential biomarker outcomes have been included. Reference to the literature can identify commercial or academic providers that can supply relevant cell types and relevant endpoints. These can be focused on developing suitable models and biomarkers to showcase the pharmacology of the candidate molecule.

These models should focus on pharmacological objectives that include a full description of the concentration response. Generally speaking, up to 10 different concentrations should ideally be investigated in order to determine an EC_50_. The EC_50_ is the concentration of the candidate molecule that affects the outcome by 50% (increase or decrease). This value is essential in the calculation of the early dose estimation and therapeutic window. It is possible that the EC_50_ value calculated in different models with different endpoints may not be identical. Therefore, it is important to study a number of different models and biomarkers in order to determine a range of EC_50_s. This range of EC_50_s can be used to estimate the likely range of exposures/doses that may be effective in subsequent clinical trials. Pharmacokinetics (PK)/pharmacodynamics (PD) modeling, together with the safety profile, is involved in determining the clinical starting dose and the maximum permitted dose in clinical trials.

**Figure 5.**
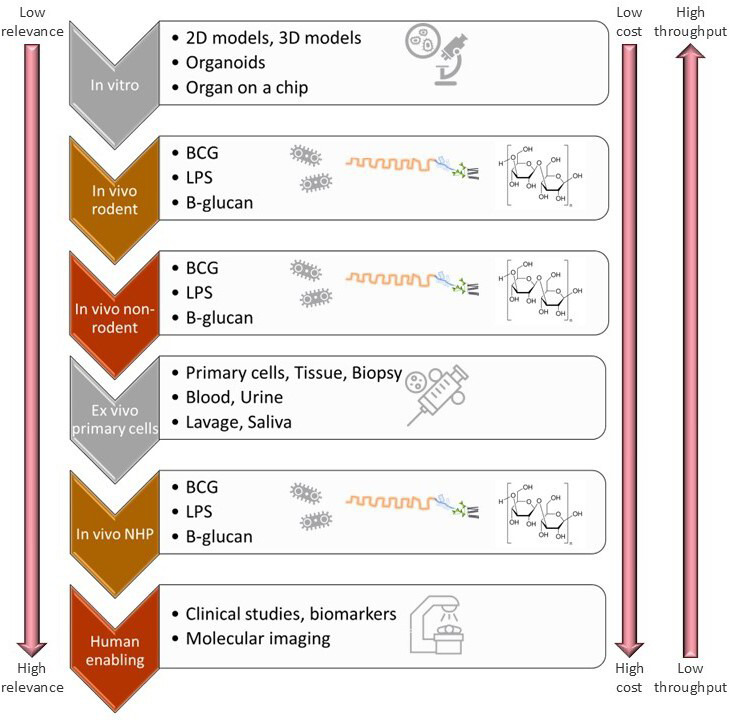
Illustration of potential types of models to assess Trained Immunity during the translational phase of drug development.

This section provides practical information that facilitates the development of suitable models and meaningful biomarkers. The types of available models are illustrated in Figure 5. The models are ranked from top to bottom, with those models that are relatively low cost and high throughput but potentially of less relevance to translational science at the top of the figure. While those with higher cost and lower throughput but of high relevance are at the bottom of the figure. During DDD, the models would be implemented over time starting with the lower cost and high-throughput type of model, and based on the outcomes of these models, the more relevant but higher cost and lower-throughput models could be defined and implemented more effectively.

The cellular components of the model are chosen to demonstrate the particular function of Trained Immunity that is of interest. These can range from cell lines, primary cells, and induced pluripotent stem cells (see Appendix 1). The process of choosing which cell lines to include in your models involves a bioinformatics exercise to investigate the expression of your drug target in the various cell lines. If a cell line does not express the molecular target of your asset, then it would be excluded from your models. In addition, some experimental work would be required to understand if the target is modulated in a particular cell line when the cells are exposed to stimulators of Trained Immunity. This evidence of modulation would prioritize this cell line for inclusion in a model.

### In vitro models

*Two-dimensional (2D) models* are considered to be the highest throughput, least expensive, but lowest physiologically relevant models. They consist of cells cultured in suspension or in a monolayer either directly onto plastic or onto plastic coated with extracellular matrix components (e.g. collagen). Adherence per se triggers various processes of differentiation which can be modulated by the stiffness of the surface to which the cells adhere. The elasticity (Young’s modulus) of most cell culture plastics is around 1 gigapascal (GPa). However, ECM stiffness of human tissue is generally lower, e.g., brain (1–3 kPa), muscle (23–42 kPa), blood vessels (1.16–860 MPa), and tendon (136–820 MPa). The exception being bone (15–40 GPa), which is actually stiffer than plastic (Yi et al., 2022). In setting up in vitro models, it may be prudent to factor in the stiffness of the matrix in the relevant tissue of interest as difficulties in inducing the cell phenotype that the model requires may occur if the cell is driven to maximum differentiation by an overly stiff matrix. A non-exhaustive table of potentially useful cell lines that are commercially available is provided in Appendix 1.

#### Examples

In vitro induction of Trained Immunity in adherent human monocytes has been described in a protocol published online (Domínguez-Andrés et al., 2021). This protocol works with adherent monocytes which are derived from peripheral blood mononuclear cells. The induction of Trained Immunity and the associated functional reprogramming of monocytes are achieved using β-glucan (from *C. albicans*) and BCG.

The HL-60 cell line is the most commonly used cell line to study human neutrophil function (Blanter et al., 2021).

- Differentiation into granulocytes: all-trans retinoic acid and dimethyl sulfoxide (DMSO) (Breitman et al., 1980; Tarella et al., 1982).
- Neutrophil functions: cell polarization, chemotaxis, reactive oxygen species (ROS), NETosis, and phagocytosis.
- Easy to culture, suitable for genetic editing

*Three-dimensional (3D) models* are more complex and more physiologically relevant than the 2D models. Cells may be embedded in a scaffolding matrix and allowed to grow in a 3D conformation. In 3D microfluidic models, cells are cultured in a tubular formation, and the culture medium is allowed to flow through the tube mimicking blood flow (Li et al., 2012). Other cell types may be added into extracellular matrix and allowed to form more complex structures.

#### Example

*Organoids* are a specific type of 3D in vitro tissue cultures. They recreate many aspects of the structure, architecture, and function of a particular in vivo tissue and can be used to understand fundamental mechanisms of Trained Immunity. They are derived from pluripotent or tissue-resident stem cells or primary differentiated cells from disease tissues. Different cell types can be combined in order to recreate the cellular population of the tissue of interest (Zhao et al., 2022). Intestinal organoids were used to investigate if Trained Immunity takes place in intestinal epithelial stem cells and how this affects their differentiation. Organoids exposed once or twice to IL-13 displayed similar properties to that of the epithelium in vivo upon a helminth infection. After a secondary challenge to the organoids, an increased goblet cell response was observed when compared to a primary one challenge. The number of goblet cells and levels of goblet-specific antimicrobial protein RELM-β were strongly increased (Levy et al., 2025).

*Organ-on-a-chip* is a technology whereby natural miniature tissues are grown inside microfluidic chips. They combine advances in tissue engineering and microfabrication to study human pathophysiology and the effect of therapeutics on the tissue. This technology in the context of Trained Immunity is discussed in more detail elsewhere (Teunissen et al., 2021).

### Ex vivo models

Fresh animal cells and tissue samples may be used ex vivo to study Trained Immunity. The use of ex vivo isolated immune cells, rather than an in vivo model, minimizes the number of animals required to conduct an experiment. It also provides translational information, if similar ex vivo samples may be collected from human participants during subsequent clinical trials. However, using ex vivo immune cells provides the opportunity to study intact human samples prior to conducting clinical trials.

The most frequently used ex vivo Trained Immunity models use primary human peripheral blood mononuclear cell (PBMC) culture or mouse spleen cells. In both cases, the ex vivo models differ from the in vitro models by the fact that the training stimulus may have been given ‘in vivo’. The PBMC culture is usually performed using 96-well round-bottom cell culture plates, whereas the monocyte-derived macrophage culture is usually performed using a 96-well flat-bottom cell culture plate (because the monocytes are adherent cells). In addition, a co-culture model that combines PBMCs with fibroblasts has been used to study Trained Immunity (Merriam and Weinberg, 2025).

Fresh human tissue (biopsy, transplant reject, tissue resection, etc.) may also be used to model Trained Immunity with the maintenance of in vivo architecture and cellular infiltrates. They can be sliced or sectioned into small pieces to be studied in tissue culture. This allows analysis of secreted proteins, cellular activation, epigenetics and metabolic changes, and immunohistochemistry. However, access to tissue may be limited, resulting in high cost and long waiting times. In addition, ex vivo human tissue will eventually deteriorate, limiting the duration of experimental procedures. It is generally not possible to study perfusion-dependent functions in this type of model.

It is possible to access human tissue directly from patients, provided that you have the ethical approval of your local ethics committee, that you obtain suitable informed consent from the donors, and that you treat the tissue according to human tissue regulations applicable in your region. Failing that, there are commercial suppliers of human tissue who will source the tissue and ensure it complies with all ethical and regulatory requirements.

### In vivo models

In vivo models of Trained Immunity can be used to study peripheral (blood), as well as central (bone marrow) Trained Immunity. Different transcriptional and epigenetic changes occur in mature innate immune cells in the periphery compared to central bone marrow progenitors of innate immune cells (Divangahi et al., 2021). Trained innate immunity provides a sustained protective response of myeloid cells to a secondary challenge, despite their short lifespan in circulation. This sustained effect has been shown to occur via modulation of hematopoetic stem and progenitor cells.

Administration of β-glucan to mice induced expansion of myeloid progenitors associated with elevated IL-1β and granulocyte-macrophage colony-stimulating factor (GM-CSF), and with changes in glucose metabolism and cholesterol biosynthesis (Mitroulis et al., 2018). The Trained Immunity-related increase in myelopoiesis resulted in a beneficial response to secondary LPS challenge.

#### Example

Introduction of BCG to the bone marrow of mice changed the transcriptional landscape of hematopoetic stem cells, leading to local cell expansion and enhanced myelopoiesis at the expense of lymphopoiesis. This generated epigenetically modified macrophages that provided significantly better protection against virulent *M. tuberculosis* infection than naïve macrophages. This reprogramming is sustainable in vivo (Kaufmann et al., 2018)*.*

A bone marrow-targeting nanobiologic (MTP_10_-HDL) was designed specifically to induce Trained Immunity and has shown potent antitumor capabilities in a mouse melanoma model. Antitumor effects resulted from Trained Immunity-induced epigenetic rewiring of multipotent progenitors in the bone marrow. These re-wired, trained cells overcame the immunosuppressive tumor microenvironment and potentiated checkpoint inhibition using anti-PD-1 and anti-CTLA-4 therapy (Priem et al., 2020).

### Biomarkers

#### General overview

**Figure 6.**
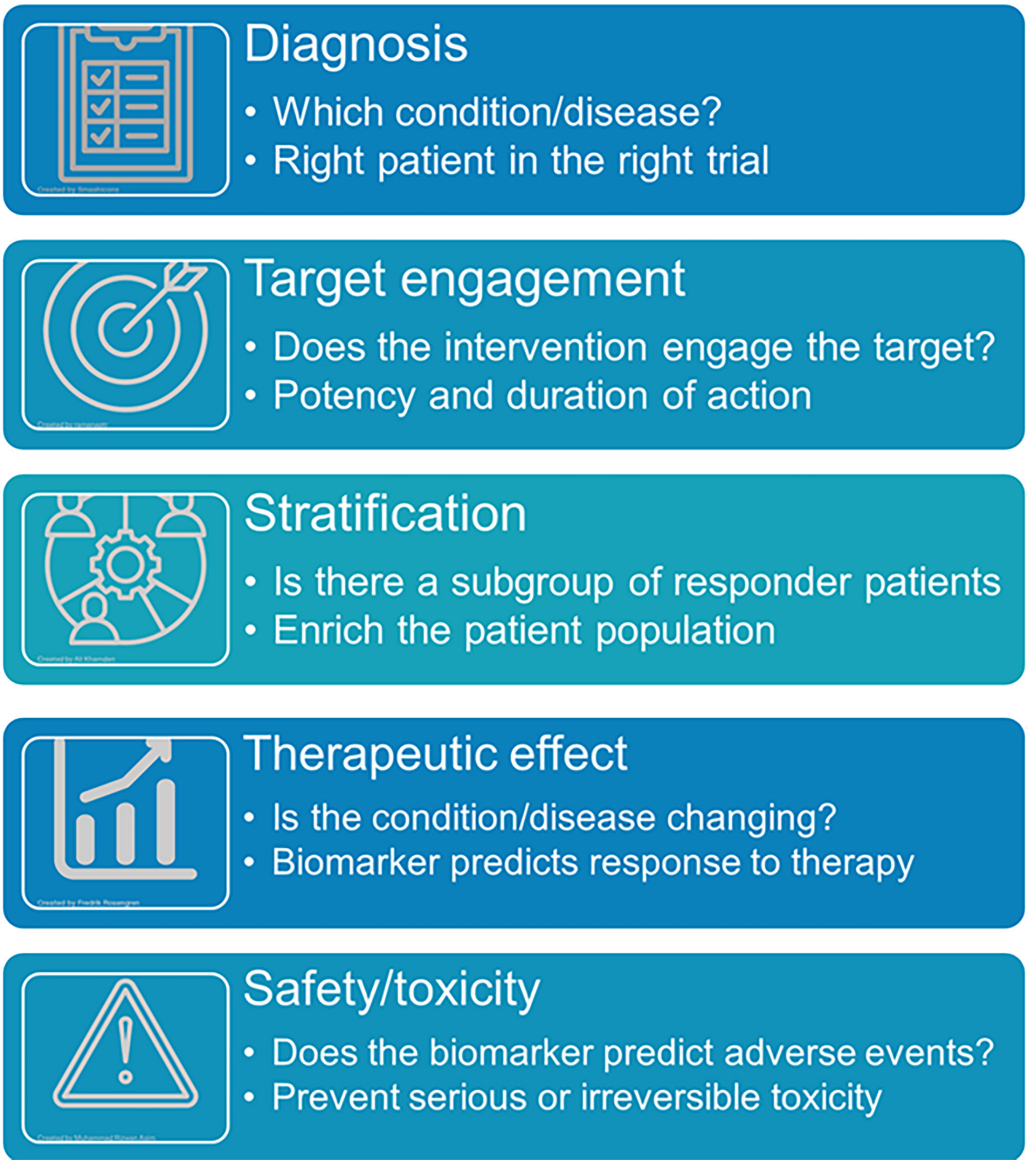
Types and applications of biomarkers (icons from the Noun project).

The definition of a ‘biomarker’ differs between experts and publications. The FDA/NIH biomarker workshop describes them as ‘defined characteristics that can be measured as an indicator of normal biological processes, pathogenic processes, or responses to an exposure or intervention, including therapeutic interventions’ (FDA-NIH Biomarker Working Group, 2016). The different types of biomarkers and their application during DDD are shown (Figure 6).

A large portion of the activities referred to as translational science involves the development of biomarkers that can be reliably applied during clinical development of a new drug. The biomarker assay needs to be robust, measurable in an accessible matrix (blood, sputum, urine, skin, etc.), have low variability, and exhibit a relationship with the biological process of interest. These biomarkers are not necessarily ‘validated’ or even ‘qualified’; they are for internal use and would not be suitable as regulatory endpoints for approval. To obtain regulatory approval for a biomarker requires a large amount of data and is beyond the scope of this review. However, the use of biomarkers in DDD is well accepted for internal decision-making.

Trained Immunity-related nonclinical models can, together with disease-specific models, provide information on the potential benefit of a candidate molecule. In addition to focusing on therapeutic benefits, it is crucial to thoroughly investigate the potential risks, as epigenetic changes may persist for an extended period and could potentially impact existing or emerging comorbidities, such as inflammation or autoimmune diseases (Divangahi et al., 2021; Bhargavi and Subbian, 2024).

The International Council for Harmonization of Technical Requirements for Pharmaceuticals for Human Use (ICH) is developing guidelines about model-informed drug development (MIDD). MIDD is defined as the strategic use of computational modeling and simulation methods that integrate nonclinical and clinical data (including biomarkers), prior information, and knowledge (e.g. drug and disease characteristics) to generate evidence (International Council for Harmonisation of Technical Requirements for Pharmaceuticals for Human Use, 2024). This initiative is groundbreaking as it allows modeling of different types of data to inform decision-making during drug development. Previously, each dataset was analyzed independently of the others, providing limited insight into safety, tolerability, and efficacy. MIDD permits a more ‘holistic’ approach that means that even preclinical and early biomarker work can still play a role at later stages in development. It enables extrapolation of that modeled data to unstudied situations and populations to mitigate risks and increase the probability of success. This has elevated the role of biomarkers in drug development from ‘nice-to-have’ to a necessity.

**Figure 7.**
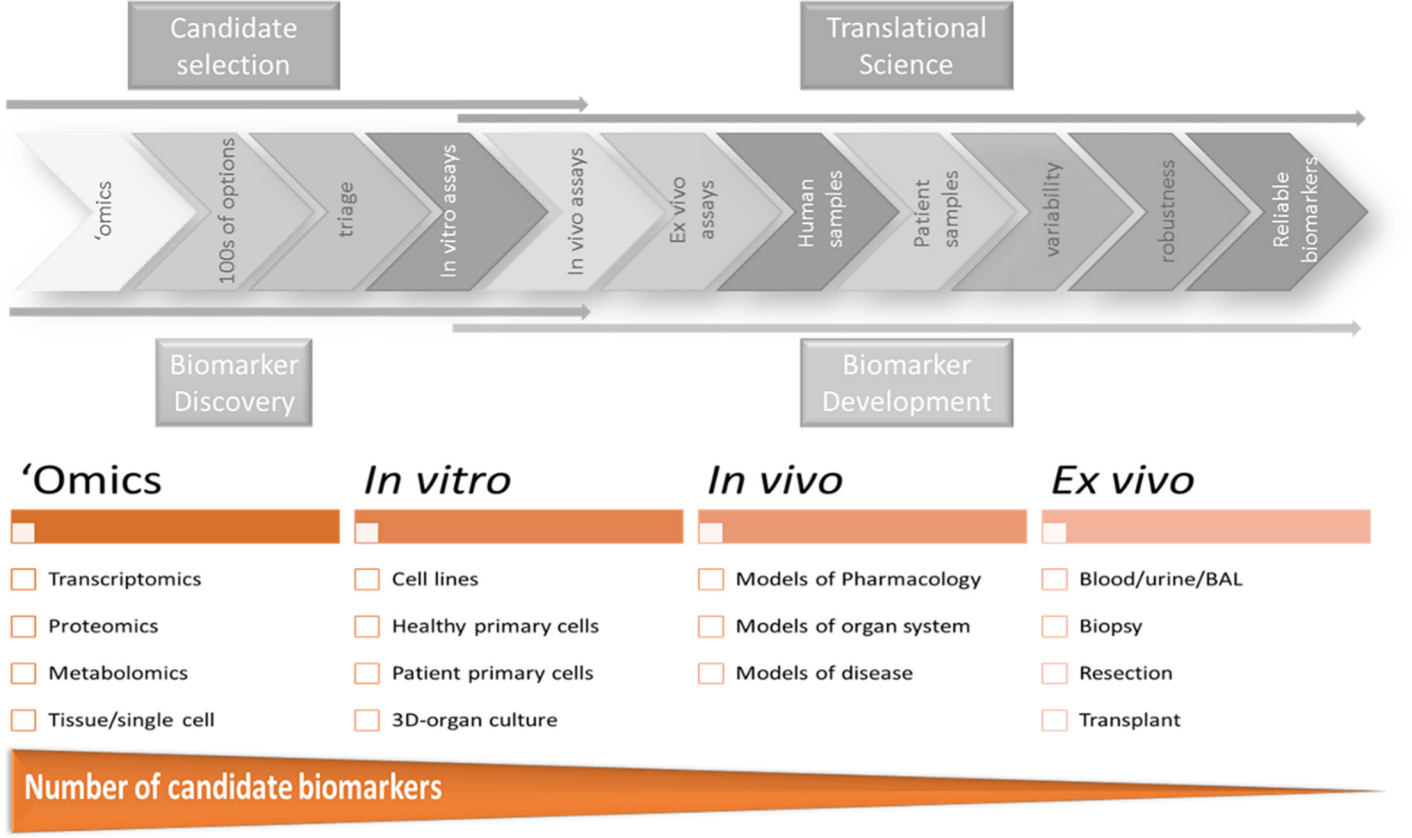
Discovery and development of biomarkers in parallel with the discovery and development of the drug.

The process for identifying potentially useful biomarkers may involve ‘omics data (transcriptomics, proteomics, etc.) generated from the relevant translational models that are conducted during the development of a new candidate (Figure 7). They could be identified as those markers that change in a dose-response manner to the pharmacological agent in a particular translational model.

### Biomarkers of Trained Immunity

#### Characterization of the models of Trained Immunity

The proposed domains of interest in the discovery and development of new therapies for Trained Immunity (Figure 2) are a good place to start thinking about biomarker discovery, using the models of Trained Immunity described above. As each model is developed, a set of biomarkers could be assayed to characterize the features of the model and answer the following questions. Which domain(s) of interest are measured in each model? How reproducible is the measurement? Which method should we use to measure the relevant biomarker? How variable is the biomarker in each model?

Once the model is well characterized, the question becomes: Does the asset under development affect the biomarker expression in a dose-dependent manner?

#### Measurement of the DDD domains of Trained Immunity in models

Biomarkers may be selected to cover these domains (Table 3).

**Table 3.** Examples of biomarkers and models of Trained Immunity.

Domains of Trained Immunity	Key markers	Assay	Context	Models	Example references
**Epigenetics**	Histone methylation H3K4me3 (active promoters), Histone acetylation H3K27ac (enhancers), H3K9me3 (repressive epigenetic marks) Chromatin accessibility	ChIP-seq, CUT&RUN, ATAC-seq, ChIP-PCR	Trained Immunity induces chromatin remodeling at cytokine and metabolic gene loci	In vitro monocyte training (e.g. with β-glucan or BCG), in vitro training of mouse bone marrow-derived macrophages	Young et al., 2011 Meers et al., 2019 Saeed et al., 2014
**Metabolic**	Glycolysis: ↑ lactate, ↑ Glut1 expression TCA cycle: Itaconate, succinate, fumarate accumulation mTOR/HIF-1α activation reactive oxygen species (ROS) NAD^+^ metabolism	Seahorse (ECAR, OCR), metabolomics (LC-MS), western blot for pathway markers HK2, LDHA (glycolysis), HIF-1α, mTOR, AMPK, SDH (succinate dehydrogenase) ROS assay	Pathways altered	In vitro Trained Immunity models with monocytes or PBMCs, both with mouse and human cells	Stefanoni, 2023 Wang et al., 2021b Mourits et al., 2021a Wu et al., 2024
**Inflammatory**	IL-6, TNF-α, IL-1β, IL-10, IL-18, IFN-γ	ELISA, Luminex, multiplex bead-based assays	Cytokine Production after stimulation with unrelated secondary ligands (e.g. LPS, Pam3CSK4), trained cells show enhanced cytokine production	In vitro monocyte and PBMC Trained Immunity assays, both with mouse and human cells. Primary human and mouse cells from in vivo studies can be used for ex vivo restimulation assays	Moorlag et al., 2020 Smith et al., 2017
**Differentiation**	Monocytes/macrophages: CD11b, CD14, CD16, CD80, CD86, HLA-DR NK cells: CD69, NKG2D, CD107a (degranulation)	Flow cytometry, mass cytometry (CyTOF)	Cell Surface and Activation Markers Trained cells may show altered expression profiles reflecting activation or increased antigen presentation	Human PBMCs, mouse peritoneal, or splenic macrophages	Gill et al., 2022 Zhang et al., 2021
**Memory**	Persistence: Epigenetic and transcriptional changes lasting weeks to months Bone marrow signatures: Myeloid progenitor reprogramming Clinical: BCG-vaccinated individuals show altered monocyte and cytokine profiles even after 3–12 months	See above examples	Longitudinal Biomarkers of Trained Immunity in in vivo studies	Ex vivo, mouse models and human models	Bomans et al., 2018 Cassone, 2018 Kuznetsova et al., 2020

##### Biomarker discovery

Transcriptomics, proteomics, and metabolomics may be used to discover de novo biomarkers related to the activity of the compound under investigation.

- **Transcriptomics:** RNA-seq of trained cells shows distinct upregulation of inflammatory and metabolic pathways (Kong et al., 2021)
- **Proteomics:** Mass spectrometry to identify Trained Immunity-related protein expression (Koeken et al., 2021)
- **Biomarkers:** Upregulation of genes like IL-1β, TNFα, NFKB1, CXCL8, SLC2A1 (Larionova et al., 2020)

### Biomarker development

Development of a translational biomarker that is suitable for internal decision-making requires painstaking assessment of the robustness, variability, and reliability of the assay in the specific matrix that will be used in the clinic (blood, biopsy, sputum, urine, etc.). It is prudent to engage with a statistician to understand the probability that significant changes in the biomarker of interest are likely to be observed during the proposed trial. Optimization of the biomarker assay and transfer of the assay to a contract research organization (CRO) that can conduct the assay during a clinical trial is an essential part of the biomarker process.

### Patient selection

AI could significantly enhance patient selection for clinical trials investigating Trained Immunity-targeting drug candidates. Furthermore, biomarker analysis as a companion diagnostic may help to select those patients who are likely to benefit from treatment, the so-called ‘responder’ population. This approach enables more targeted and personalized therapeutic interventions. Screening for the pretreatment immune status of patients can enhance patient selection and optimize the use of Trained Immunity-regulating compounds.

In case of an indication where Trained Immunity should be inhibited, screening for pretreatment immune status can identify which immune pathway or process is dysregulated. Next, the most appropriate target and Trained Immunity-regulating compound targeting this specific pathway or process can be selected.

In case of an indication where Trained Immunity responses should be activated or induced, screening for baseline immune status can potentially also inform which patient will benefit the most. For example, researchers observed that the induction of Trained Immunity after BCG vaccination was most effective in individuals with a dormant pretreatment immune status. Specifically, the researchers showed that there were specific chromatin regions with different accessibility before treatment which could be used to differentiate the good from the poor Trained Immunity responders (Moorlag et al., 2024). It can be hypothesized that screening for such chromatin regions prior to treatment can identify good responders to Trained Immunity before treatment initiation. Patients with inflammatory comorbidities may potentially be selected for Trained Immunity-inhibiting compounds. It has been shown experimentally that periodontal disease and arthritis in mice are characterized by maladaptive Trained Immunity responses (Li et al., 2022). Whether this phenotype is also present in humans and whether it can be effectively targeted by Trained Immunity inhibitors remain to be elucidated.

## Translational pathway

**Figure 8.**
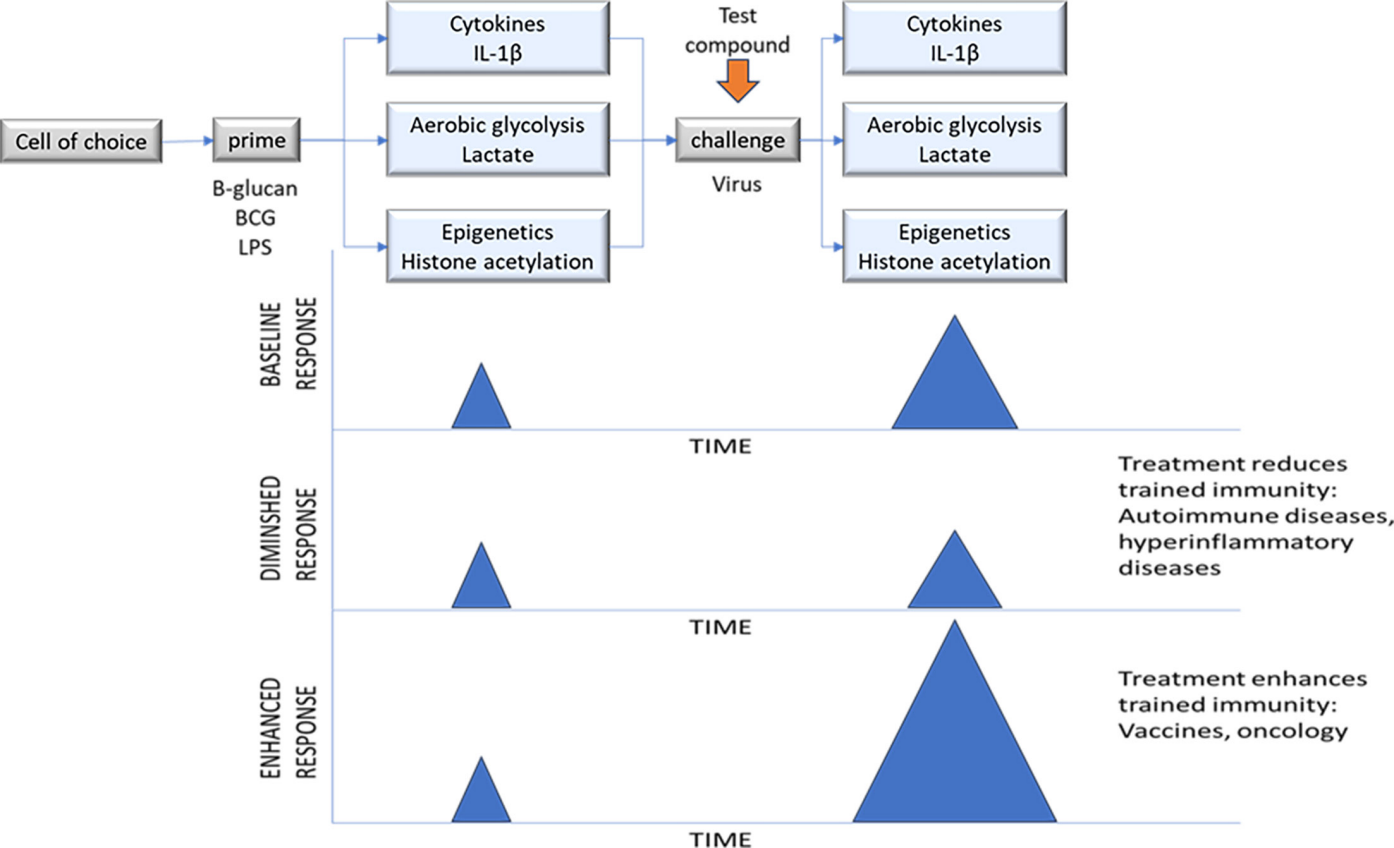
Overview of a possible in vitro study design to investigate the activity of a test compound in a model of Trained Immunity. A stimulus to prime innate immunity may be added to the model and endpoints measured (cytokines, metabolism, and epigenetics). Over time, the effect of the priming stimulus fades and most of the endpoints return to baseline levels (the exception being epigenetics, where the modifications may be longer-lived). The test compound may be added at various concentrations at the same time as the secondary challenge. Subsequent effects on cytokines, metabolism, and epigenetics may be measured. The basal response (measured in the absence of treatment) may be compared to the posttreatment response. The compound may decrease the trained immune response, or it may augment the response.

An important step is to develop and validate a suitable model that expresses all of the features of Trained Immunity that may be affected by your new candidate (based on the known molecular target and/or the phenotypic screen). A good starting point could be an in vitro model using a cell line. This would allow a preliminary characterization of the potential pharmacology of the candidate. A schematic is shown in Figure 8. In this experimental design, a suitable cell line would be chosen based on a bioinformatic investigation to measure levels of expression of the molecular target. The cell line with the appropriate level of expression could be cultured in vitro and differentiated into the cell type of interest (e.g. HL-60 cells differentiated into granulocytes). These cells could then be primed using the known initiators of Trained Immunity (β-glucan, BCG, LPS). Increases in the relevant domains of DDD in Trained Immunity could be assessed in the model. The time required for these upregulated analytes to return to baseline values could be determined. Once the analytes have returned to baseline, the secondary challenge (e.g. virus) could be applied and the memory increase in Trained Immunity could be assessed. This model should be validated by showing that the data generated is robust, reproducible, and reliable.

Thinking of the timing for adding the candidate molecule to the model depends on the desired product profile of the drug under development and its ultimate planned use in the clinic. It is likely that a modulator of Trained Immunity would be administered after the initial prime has taken place but before/during the secondary challenge. In the example below (Figure 8), the candidate is added at the time of the secondary challenge; however, this could be varied based on emerging data. The effect of the candidate on the trained immune response can then be assessed. The candidate may enhance or diminish all of the aspects of Trained Immunity, or it may provide a more complex pattern of effect with some analytes enhanced and others diminished.

Comprehensive dose-response data should be provided for each of the endpoints measured in the model, such that an EC_50_ can be calculated for each endpoint (Sebaugh, 2011).

The translational value of a model is somewhat difficult to assess, particularly in the field of Trained Immunity where you may be developing a treatment to increase Trained Immunity or a treatment to decrease Trained Immunity. In addition, the timing of the first application of your asset into the model is also crucial. Thinking about the development of a vaccine adjuvant (Figure 8), the asset would be most appropriately added prior to the priming event, as this would mimic the clinical situation. However, in a complex waxing and waning systemic disease like rheumatoid arthritis, when is it most appropriate to administer the asset to the model? One could argue that the priming event occurred long before diagnosis, and the disease is now driven by self-sustaining immune responses. In this case, the asset could be administered during the challenge or even later at peak effects. Alternatively, one could argue that the priming event is repeated continuously with generation of Trained Immunity as an ongoing cycle of priming and challenge. In this case, the administration of the asset at or before priming could still be relevant.

In conclusion, the design of the model used to test your asset and determine its pharmacology should be guided by the proposed indication and use in the clinic. This is the target product profile – a description of the use and expected effects of your molecule in a particular patient population.

## Indications

Based on the mechanism of action (a memory-like response after exposure to certain stimuli, leading to enhanced responsiveness to future infections) and the cells involved (macrophages, NK cells, monocytes, etc.), Trained Immunity offers promising applications across various medical conditions, primarily in enhancing host defense against infections and modulating chronic inflammatory diseases.

It is tempting to think of separating indications into those that require Trained Immunity to be augmented and those that require a decrease in Trained Immunity. However, the reality is likely to be more nuanced, with each indication requiring modulation of Trained Immunity with some aspects being augmented and others decreased. This emphasizes the need to understand the pharmacology of a new asset and its overall effect on the various models of Trained Immunity. This information can then be used to find the patient population that is most likely to derive benefit from the new therapeutic agent.

AI drug positioning engines can screen multiple disease indications and rank them based on their relevance to specific molecular targets or pathways involved in Trained Immunity. This approach can help prioritize the most promising indications for further investigation.

Here are some of the most promising indications for Trained Immunity.

### Infectious diseases

- BCG and other vaccinations, in particular as an adjuvant: There is a growing body of evidence that the BCG vaccine is capable of inducing Trained Immunity, providing protection not only against tuberculosis but also against heterologous infections, including viral respiratory tract infections (Netea et al., 2020b) and fungal infections as well. Numerous publications and research activities around the BCG vaccination confirm this observation initiated by Swedish pediatricians about 100 years ago. Based on this knowledge, the development of an adjuvant in combination with an antigen, or an immune stimulant which is pathogen-agnostic, appears so far as the most promising use of Trained Immunity.
- Nosocomial infections: Trained Immunity-based vaccines can offer rapid protection against antibiotic-resistant bacteria like *Acinetobacter baumannii* and *Pseudomonas aeruginosa*, which are significant threats in healthcare settings. Antimicrobial resistance is already highly relevant to healthcare today and will likely be even more so in the future. Trained Immunity could play both prophylactic and therapeutic roles in this indication. (Mucosal) Trained Immunity-based vaccines may present a promising approach to reduce the burden of antimicrobial resistance (Minute et al., 2025).
- Fungal infections: Potential applications include developing vaccines that protect against fungal infections, which could be beneficial in preventing complications during surgery or in inflammatory bowel disease (Dagenais et al., 2023).

### Chronic inflammatory conditions

- Atherosclerosis and atherosclerotic cardiovascular disease: Trained Immunity plays a role in the pathophysiology of atherosclerosis and cardiovascular disease (Flores-Gomez et al., 2021; Chen et al., 2025; Bahrar et al., 2024). Modulating Trained Immunity could have beneficial effects in managing chronic inflammation associated with this condition (Tercan et al., 2021).
- Transplant rejection: Understanding Trained Immunity can help in developing strategies to prevent transplant rejection by modulating the immune response (Tercan et al., 2021).

### Immunocompromised individuals

- Trained Immunity-based vaccines can provide broad protection, which is particularly advantageous for immunocompromised individuals who may not respond well to traditional vaccines (Dagenais et al., 2023). Strengthening the immune system of the host is a relevant means for the treatment of solid tumors in general, but in immunosuppressed patients in particular.

### Neuroinflammatory disorders and autoimmune diseases

- Trained Immunity can be modulated to treat neuroinflammatory disorders and autoimmune diseases by either stimulating or inhibiting immune responses (Bindu et al., 2022).
- Systemic lupus erythematosus (SLE): It is hypothesized that Trained Immunity may be one of the mechanisms involved in SLE (Mora et al., 2023). Accordingly, the cytokine profile of SLE is similar to the cytokine profile induced during Trained Immunity (Funes et al., 2022). Furthermore, IL-1β is a key cytokine driving SLE (Caielli et al., 2024; Italiani et al., 2018) and also plays a central role during Trained Immunity. Therefore, inhibiting Trained Immunity in SLE could potentially reduce the severity of the systemic inflammation.
- Rheumatoid arthritis (RA): The increased levels of IL-1β, TNFα, and IL-6 which are found in RA also resemble a Trained Immunity phenotype. Additionally, the monocytes isolated from inflamed joints of RA patients are more prone to metabolic reprogramming and display enhanced glycolysis. These metabolic changes resemble a Trained Immunity phenotype, and it can be hypothesized that Trained Immunity plays a role in the pathophysiology of RA (Mora et al., 2023; Badii et al., 2022).
- Gout: The pathophysiology of gout evolves around urate crystals which trigger acute gout flares, characterized by inflammasome activation and cytokine production. It has been shown that urate crystals can induce epigenetic reprogramming of monocytes, and Trained Immunity has been proposed as one of the mechanisms of gout, which could potentially be targeted (Badii et al., 2022; Straton et al., 2024; Gaal et al., 2025).

### Oncology, in particular treatment of solid tumors

- The observation that patients with tuberculosis have a lower incidence of tumors led to the research of BCG as an antitumor treatment, which was among others demonstrated in the indication ‘bladder cancer’. Antitumor activity could be demonstrated for BCG and other stimulants of the innate immunity like Imiquimod (Singh et al., 2021; van Puffelen et al., 2023; Sui and Berzofsky, 2024).

The development of treatment modalities utilizing Trained Immunity as a mechanism of action has just begun. Many questions remain to be addressed. The most obvious indication is in infectious diseases with a growing body of data supporting it. Cancer, immunocompromised patients, and autoimmune diseases are of interest as well.

### Entering the development phase

Once the discovery process is completed and the product development process starts, regulatory guidance becomes increasingly important, to discuss nonclinical proof of concept or the selection of the species for nonclinical testing. Specifically, Trained Immunity-related Guidance documents are not (yet) available. As with other newly arising areas of drug discovery, it is advisable to ask FDA for pre-IND meetings and Scientific Advice meetings with Regulatory Agencies such as those in Europe.

## Clinical trials

In drug development, clinical trials are the point at which a drug is tested in humans with the purpose of establishing safety and efficacy in the chosen indication(s). Arrival at this point in development brings additional ethical, financial, medical, and scientific considerations that need to be carefully considered to design studies capable of demonstrating whether or not the drug works while limiting the risk to the health of the participants (FDA, 2018). As discussed in Appendix 1, clinical trials are typically classified into phases; the most relevant of which for development of a new drug are Phase 1, Phase 2, and Phase 3. Trials may be classified as multiple phases if relevant.

### Phase 1

Phase 1 refers typically to clinical trials primarily aiming to establish safety and tolerability of doses of a drug. These trials are typically conducted in a limited number of participants (typically 20–100), usually healthy participants, although sometimes patients are recruited when drugs are expected to be unsafe in healthy participants, e.g., for cancer chemotherapeutics with expected side effects (FDA, 2018).

Phase 1 may be further subdivided into Phases 1a and 1b. Phase 1a typically refers to a Single Ascending Dose trial where groups of participants receive a single dose of the compound or placebo and are monitored for a period of time. If the dose of the compound is safe and tolerated, then the dose level is increased (escalated) and administered to a new group of participants. Dose escalation is stopped either when intolerable adverse events occur, establishing a maximum tolerated dose, or when a predetermined exposure limit has been reached. The Single Ascending Dose trial is followed by a Multiple Ascending Dose trial where participants are administered multiple doses of the compound or placebo at intervals. As with a Single Ascending Dose trial, the dose levels are increased for subsequent groups of participants until stopped by reaching a predetermined limit or stopped due to adverse events.

Phase 1b refers to early exploratory trials in patients with the indication of interest. The objective of Phase 1b trials is to investigate the safety, tolerability, PK, and PD of the candidate drug in a patient population. This type of study is sometimes referred to as Proof of Pharmacology.

Although preclinical work in model systems can be used to estimate what doses of a new drug are likely to be tolerated and what doses are likely required to be efficacious, these methods remain inherently limited. A First-In-Human (FIH) trial thus has the inherent risks that the human response to the drug is unknown until tried. Accordingly, trial designs must acknowledge and attempt to minimize this risk, e.g., through the practice of ‘sentinel dosing’ where the first time a new dose level is tested, the drug or placebo is administered to a limited number of participants (e.g. two participants, one randomized to active and one to placebo) until it has proven sufficiently safe to test in further participants.

While the primary outcome or endpoint of a Phase 1 trial is typically safety and tolerability, secondary outcomes or endpoints may involve assays to measure target engagement, PD, and PK that may be used to inform the design of a Phase 2 trial.

### Phase 2

A Phase 1 trial should deliver sufficient data to establish the safety of testing the drug in humans, but typically will not be able to demonstrate whether the drug is efficacious in the chosen indication(s), either simply because the drug was only tested in healthy volunteers or because the trial was not statistically powered to demonstrate a treatment effect in the patient population. A Phase 2 trial, therefore, attempts to demonstrate whether the drug has a clinically relevant effect on patients. Phase 2 trials are larger (typically several hundred participants) than Phase 1 and may be the first time a drug is tested in a patient population rather than healthy volunteers (FDA, 2018). As with Phase 1 trials, Phase 2 trials may be subdivided into Phase 2a and 2b.

Phase 2a generally refers to a trial establishing initial clinical proof of concept where a clinically relevant patient response to an estimated therapeutic dose of the drug is monitored. Phase 2b generally refers to larger dose-ranging trials, where multiple doses are tested to inform the dose to be tested in a Phase 3 trial.

### Phase 3

Phase 3 is the final phase of clinical development before a drug may be considered for approval by medical regulators. They are the largest of the three phases (typically 300–3000 participants) and are the most expensive (FDA, 2018). The decision to proceed to a Phase 3 trial must thus be based on promising results from Phase 2 trials. A Phase 3 trial aims to test the drug in a large population of patients and compare it to the current standard of treatment. This should establish whether the new drug works in a clinical setting and what, if any, benefits it has compared to the current standard of treatment.

### Other phases

Early Phase 1 is a classification used for trials more limited than a traditional Phase 1. Phase 4 trials are trials of drugs that have already been approved in one indication but are undergoing further study, for example seeking to obtain additional claims on their label. Life cycle management of an approved drug generally involves Phase 4 trials.

### Clinical trials and Trained Immunity

The concept of Trained Immunity was defined, relatively recently, in 2011, and translation of these findings into clinical use is still somewhat limited. Accordingly, while there are numerous novel therapies aiming to influence Trained Immunity under investigation, the available clinical trial data is dominated by investigation of already developed or approved therapies, particularly vaccines such as the BCG vaccine and the MV130 vaccine (Wilkie et al., 2022; Montalbán-Hernández et al., 2024).

**Figure 9.**
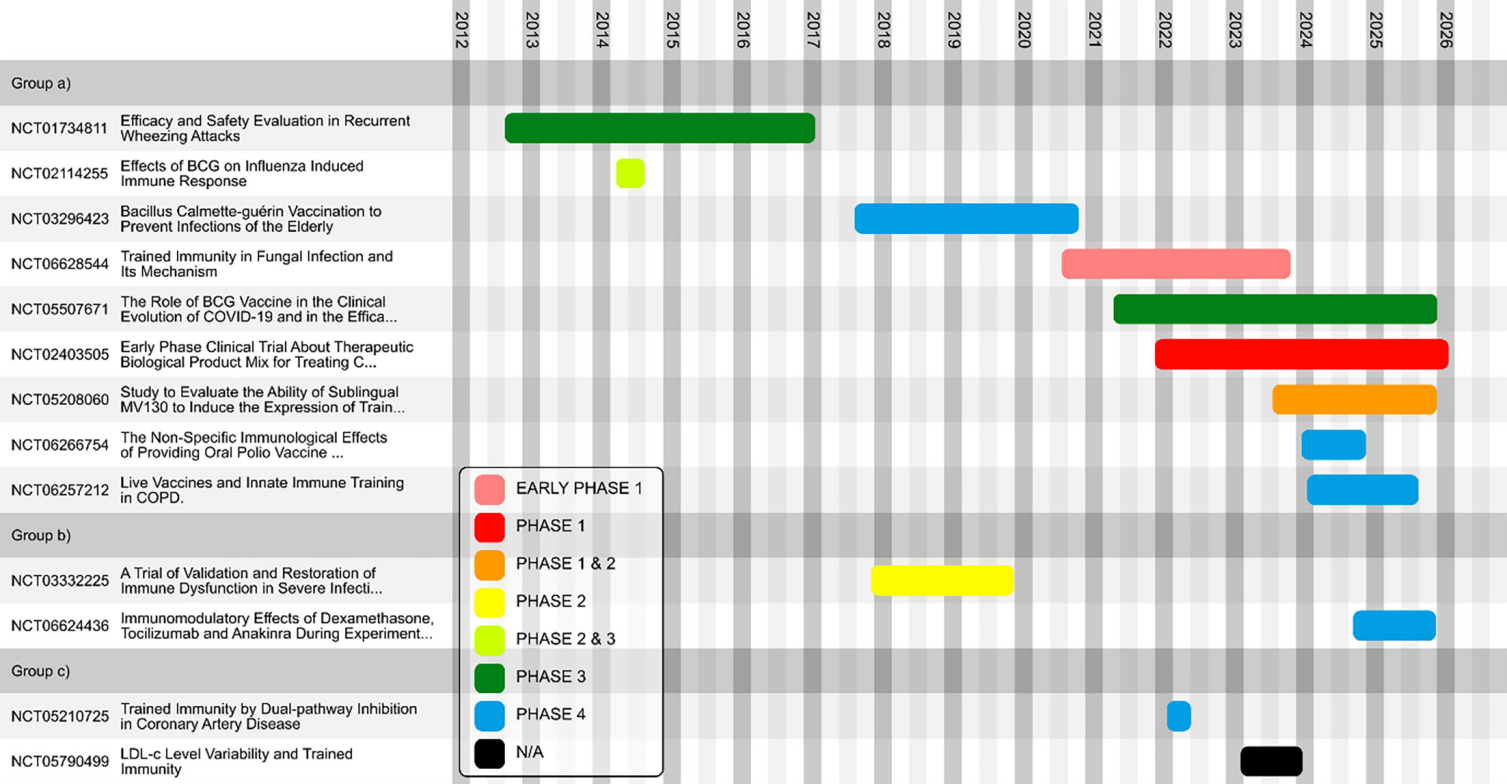
Selected clinical trials investigating the modification and regulation of Trained Immunity. (**a**) Trials investigating induction of Trained Immunity for therapeutic benefit, (**b**) trials investigating modulation of Trained Immunity for therapeutic benefit, and (**c**) trials investigating inhibition of Trained Immunity for therapeutic benefit.

Thus, there is not a defined clinical path to the market for novel assets based upon clear clinical trial precedents of drugs approved on the basis of their modification of Trained Immunity. Nonetheless, there are clinical trials that have examined the response of the innate immune system to interventions. Included in this section are several clinical trials that have been selected to inform on how to proceed through clinical development with drugs targeting Trained Immunity (Figure 9).

These trials are categorized into three groups:

- trials investigating induction of Trained Immunity for therapeutic benefit,
- trials investigating modulation of Trained Immunity for therapeutic benefit, and
- trials investigating inhibition of Trained Immunity for therapeutic benefit.

The examples given in group (a) are primarily investigating vaccines, particularly the BCG vaccine, and whether they confer protection from diseases other than the typical target of the vaccine, by inducing a Trained Immunity effect.

Group (b) includes two trials investigating whether immunomodulatory drugs can alter features of sepsis and experimental endotoxemia such as immunoparalysis. These trials provide interesting examples of how modulating immune responses, e.g., with Anakinra (recombinant IL-1R antagonist), to achieve therapeutic goals relevant to Trained Immunity can be tested in human participants.

Group (c) includes two trials where different treatment regimens, dual versus single pathway inhibition in coronary artery disease and continuous versus intermittent statin treatment for control of cholesterol, are compared and investigated for their effects upon Trained Immunity in the context of cardiovascular health. These trials thus demonstrate examples for how inhibiting maladaptive Trained Immunity responses may be investigated.

The conduct of these trials over time is illustrated by Phase (Figure 9). While the earliest trial selected began in 2012, most of the trials selected are more recent.

More details of the individual trials show the various endpoints that are included in the trials in order to showcase the effect of the investigational molecule on Trained Immunity in humans (Table 4).

**Table 4.** Selected clinical trials investigating interventions in the context of Trained Immunity.

(a) Trials investigating induction of Trained Immunity for therapeutic benefit		
**1**	**NCT ID**	NCT06257212
	**Title**	Live Vaccines and Innate Immunity Training in COPD
	**Dates**	2024/02/28 to 2025/09
	**Phase**	Phase 4
	**Enrolment**	60 (Estimated)
	**Condition(s)**	COPD
	**Intervention(s)**	BCG vaccine MMR vaccine
	**Primary Outcome**	Innate immune training measured by fold-changes in cytokine production capacity of innate immune cells following pro-inflammatory stimulation. Measured from inclusion in the trial to 4 months’ post-inclusion. Cytokines include: IL-1β, IL-10, TNF-α, IFN-γ
**2**	**NCT ID**	NCT06266754
	**Title**	The Non-Specific Immunological Effects of Providing Oral Polio Vaccine to Seniors in Guinea-Bissau
	**Dates**	2024/01/29 to 2024/12/31
	**Phase**	Phase 4
	**Enrolment**	80 (Estimated)
	**Condition(s)**	Vaccine Reaction
	**Intervention(s)**	Oral Polio vaccine
	**Primary Outcome**	Levels of proinflammatory cytokines (including IL1-β, TNF-α, IFN-γ) after stimulation of PBMCs with non-OPV antigens and mitogens 1 month after intervention Levels of plasma markers of systemic inflammation (e.g. TWEAK and SIRT2) 1 month after intervention Investigating epigenetic changes in PBMCs by single-cell ATAC-sequencing and whole-genome methylation assays 1 month after intervention Investigate transcriptional effects on immune cells by single-cell RNA-sequencing 1 month after intervention. Identifying proportions of immune cell subsets
**3**	**NCT ID**	NCT05208060
	**Title**	Study to Evaluate the Ability of Sublingual MV130 to Induce the Expression of Trained Immunity in Peripheral Blood Cells
	**Dates**	2023/09/01 to 2025/12/31
	**Phase**	Phases 1 and 2
	**Enrolment**	48 (Estimated)
	**Condition(s)**	Immune Response
	**Intervention(s)**	MV130 vaccine
	**Primary Outcome**	Increase in ex vivo PBMCs cytokine response (TNF-α, IL-6, IL-1β) to secondary restimulation compared to placebo at days 15, 45, and 70 with respect to baseline
	**Selected Secondary Outcomes relevant to Trained Immunity**	Epigenetic and metabolic changes in purified monocytes from PBMCs, including specific Trained Immunity-associated miRNAs (miR155, miR146, miR21), lactate production, glucose consumption, and mitochondrial activity at day 45 with respect to baseline Change in proportions of immune cells (including T cells, B cells, NK cells, and subsets of monocytes) in peripheral blood at days 15, 45, and 70 with respect to baseline
**4**	**NCT ID**	NCT02403505
	**Title**	Early Phase Clinical Trial About Therapeutic Biological Product Mix for Treating CEA Positive Rectal Cancer
	**Dates**	2021/12/28 to 2025/02/28
	**Phase**	Phase 1
	**Enrolment**	20 (Estimated)
	**Condition(s)**	Rectal Cancer
	**Intervention(s)**	CEA protein antigen and BCG vaccine mix for percutaneous use
	**Primary Outcome**	Timeframe: up to 90 days Participants with positive CEA blood test Participants with positive IGRA blood test with CEA protein antigen after percutaneous use Participants with IGRA blood test with TB antigens (negative before percutaneous use, positive after percutaneous use)
**5**	**NCT ID**	NCT05507671
	**Title**	The Role of BCG Vaccine in the Clinical Evolution of COVID-19 and in the Efficacy of Anti-SARS-CoV-2 Vaccines
	**Dates**	2021/05/27 to 2023/12/31
	**Phase**	Phase 3
	**Enrolment**	556 (Estimated)
	**Condition(s)**	COVID-19
	**Intervention(s)**	BCG vaccine
	**Primary Outcome**	Incidence of SARS-CoV-2 infection. Timeframe: 6 months from recruitment day Incidence of COVID-19 symptoms. Timeframe: 6 months from recruitment day Intensity of efficacy of first dose of vaccine against COVID-19. Timeframe: 6 months from recruitment day Duration of efficacy of the second vaccine dose against COVID-19. Timeframe: 1 year from recruitment day
	**Selected Secondary Outcomes relevant to Trained Immunity**	Serum concentrations of cytokines TNF-α, IFN-γ, IL-1β, IL-4, IL-6, and IL-10 in 50 participants of BCG group versus 50 participants of placebo group 2 months after recruitment
**6**	**NCT ID**	NCT06628544
	**Title**	Trained Immunity in Fungal Infection and Its Mechanism
	**Dates**	2020/09/01 to 2023/12/01
	**Phase**	Early Phase 1
	**Enrolment**	79 (Actual)
	**Condition(s)**	BCG vaccination
	**Intervention(s)**	BCG vaccine Metformin
	**Primary Outcome**	IL-6 and TNF-α cytokine production by PBMCs isolated after 5 days of continuous medication and restimulated with *C. albicans* or *Mycobacterium tuberculosis*
**7**	**NCT ID**	NCT03296423
	**Title**	Bacillus Calmette-Guérin Vaccination to Prevent Infections of the Elderly
	**Dates**	2017/09/21 to 2020/11/30
	**Phase**	Phase 4
	**Enrollment**	200 (Actual)
	**Condition(s)**	Infection Hospitalization Mortality
	**Intervention(s)**	BCG vaccine
	**Primary Outcome**	Time to first infection. Timeframe: 12 months
	**Selected Secondary Outcomes relevant to Trained Immunity**	Cytokine stimulation from PBMCs. Timeframe: month 3 Epigenetic changes of circulating monocytes. Timeframe: month 3
**8**	**NCT ID**	NCT02114255
	**Title**	Effects of BCG on Influenza Induced Immune Response
	**Dates**	2014/05 to 2014/09
	**Phase**	Phases 2 and 3
	**Enrolment**	40 (Actual)
	**Condition(s)**	Influenza virus infection Trained Immunity
	**Intervention(s)**	BCG vaccine
	**Primary Outcome**	Difference in influenza antibody titers at days 14, 21, 28, and 42 Difference in thrombocyte function at days 0, 14, 21, 28, and 42
	**Selected Secondary Outcomes relevant to Trained Immunity**	IFN-γ, IL-10, type 1 IFN, IL-17, IL-22 production by ex vivo leukocytes stimulated with inactivated/live influenza virus at days 0, 14, 28, and 42 Production of inflammatory mediators (including TNFα, IL-1β, IFN-γ, IL-10, IL-17, and IL-22) by ex vivo leukocytes stimulated with different stimuli (including *M. tuberculosis*, *S. aureus*, *C. albicans,* and inactivated influenza) at days 0, 21, 28, and 42 qPCR/microarray of inflammatory transcriptional pathways at days 0, 14, 21, 28, and 42. Granzyme B production by ex vivo leukocytes stimulated with inactivated/live influenza virus at days 0, 14, 21, 28, and 42
**9**	**NCT ID**	NCT01734811
	**Title**	Efficacy and Safety Evaluation in Recurrent Wheezing Attacks (MV130)
	**Dates**	2012/10 to 2017/02
	**Phase**	Phase 3
	**Enrolment**	120 (Actual)
	**Condition(s)**	Bronchospasm Bronchiolitis Bronchitis
	**Intervention(s)**	MV130 vaccine
	**Primary Outcome**	Number of Recurrent Bronchospasm (Wheezing Attacks)
(b) Trials investigating modulation of Trained Immunity for therapeutic benefit		
**10**	**NCT ID**	NCT06624436
	**Title**	Immunomodulatory Effects of Dexamethasone, Tocilizumab and Anakinra During Experimental Human Endotoxemia
	**Dates**	2024/10/24 to 2025/12
	**Phase**	Phase 4
	**Enrolment**	52 (Estimated)
	**Condition(s)**	Sepsis Neuroinflammatory Response Immunosuppression Endotoxemia
	**Intervention(s)**	Dexamethasone Tocilizumab Anakinra
	**Primary Outcome**	Plasma TNF concentrations upon second LPS challenge Cerebrospinal fluid TNF concentrations during repeated experimental human endotoxemia
	**Selected Secondary Outcomes relevant to Trained Immunity**	Plasma cytokine (IL1RA, IL-6, IL-8, IL-10, MIP-1α, MIP-1β, MCP-1, G-CSF, IP-10, CX3CL1, YKL-40) concentrations (plasma and cerebrospinal fluid), other inflammatory protein biomarkers (Olink Target 96 inflammation panel) (plasma and cerebrospinal fluid), and mHLA-DR during first and second LPS challenges Blood leukocyte single-cell and bulk mRNA profiles/transcriptomic pathways upon LPS challenges Cytokine production of ex vivo leukocyte cultures
**11**	**NCT ID**	NCT03332225
	**Title**	A Trial of Validation and Restoration of Immune Dysfunction in Severe Infections and Sepsis
	**Dates**	2017/12/15 to 2019/12/31
	**Phase**	Phase 2
	**Enrolment**	36 (Actual)
	**Condition(s)**	Sepsis Macrophage Activation Syndrome
	**Intervention(s)**	Anakinra Recombinant human IFN-γ
	**Primary Outcome**	Mortality. Timeframe: 28 days
	**Selected Secondary Outcomes relevant to Trained Immunity**	Cytokine stimulation from PBMCs. Timeframe: 4 and 7 days Gene expression of PBMCs. Timeframe: 7 days Epigenetic changes of circulating monocytes. Timeframe: 7 days
(c) Trials investigating inhibition of Trained Immunity for therapeutic benefit		
**12**	**NCT ID**	NCT05790499
	**Title**	LDL-c Level Variability and Trained Immunity
	**Dates**	2023/03/20 to 2024/01/31
	**Phase**	N/A
	**Enrollment**	12 (Estimated)
	**Condition(s)**	Cholesterol Variability Trained Immunity
	**Intervention(s)**	Atorvastatin
	**Primary Outcome**	Changes in LDL-C levels between baseline and atorvastatin treatment cycles. Timeframe: 16 weeks
	**Selected Secondary Outcomes relevant to Trained Immunity**	Timeframe: 16 weeks PBMCs subgroup percentage and activation status PBMCs secreting cytokines PBMCs change in gene expression Levels of hs-CRP, IL-6, IL-18, and sVCAM-1
**13**	**NCT ID**	NCT05210725
	**Title**	Trained Immunity by Dual-pathway Inhibition in Coronary Artery Disease
	**Dates**	2022/03/01 to 2022/07/01
	**Phase**	Phase 4
	**Enrolment**	20 (Actual)
	**Condition(s)**	Coronary Artery Disease
	**Intervention(s)**	Rivaroxaban and Acetylsalicylic acid
	**Primary Outcome**	Whole blood immune responsiveness to LPS stimulation when switching from acetylsalicylic acid monotherapy to acetylsalicylic acid and low-dose rivaroxaban dual pathway inhibition. Timeframe: 12 weeks
	**Selected trial outcomes relevant to Trained Immunity**	White blood cell count and distribution. Timeframe: 3 months Monocyte immune responsiveness to LPS stimulation. Timeframe: 3 months Enrichment of epigenetic gene marks. Timeframe: 3 months

Table of examples of interventional clinical trials related to Trained Immunity. Primary and secondary outcome fields have been simplified from the original data. Only secondary outcomes related to Trained Immunity are included. Source: https://clinicaltrials.gov/.

### What can we learn from past clinical trials?

The design of clinical trials must be carefully considered to ensure the trial is able to answer the questions it aims to address. These objectives differ across the phases, with phase 1, for example, primarily aiming to demonstrate safety and tolerability but also providing an opportunity to examine whether a drug actually engages with the expected target. Phase 3 trials, in contrast, are focused on confirming clinical benefit, and so the primary outcome/endpoint for these trials will be a measure of clinical benefit, likely specific to the indication(s) included in the participant population. Thus, it is important to design trials with appropriate outcomes or endpoints. Furthermore, it is important to design trials with sufficient statistical power to discern the effect of an intervention upon these outcome measures, e.g., planning a sufficiently large trial enrollment of a patient population for the expected effect size.

From the examples of clinical trials in Table 4, we can see there are a number of outcomes that may be used in assessing drugs with regard to Trained Immunity. We can examine these with respect to the drug development domains of Trained Immunity.

#### Epigenetic

Epigenetics changes are examined in trials NCT06266754, NCT05208060, NCT05790499, NCT05210725, NCT03296423, NCT0333225. These outcomes include direct measures of DNA and chromatin structure such as single-cell ATAC-sequencing and whole-genome methylation assays. Other measures look at examining gene expression changes and investigating changes in specific Trained Immunity-associated miRNAs. Different trials report different cell populations as being of interest for their epigenetic changes; for instance, some specify monocytes while others more generally refer to PBMCs.

#### Metabolic

NCT05208060 measures metabolic changes in purified monocytes by examining lactate production, glucose production, and mitochondrial activity.

#### Differentiation

Changes in differentiation may be measured simply by examining the proportions of immune cells in peripheral blood, as trial NCT05208060 does. Another example of examining the changes in differentiation is trial NCT05790499 measuring PBMC subgroup percentages and activation status.

#### Inflammation

Inflammation-related outcomes are common across many trials, being a relatively straightforward way of measuring the activity of the innate immune system and how it changes upon intervention. However, there are different ways to interrogate this. Some outcomes measure serum or plasma concentrations of cytokines such as TNF-α, IL-1β, and IFN-γ, i.e., in vivo measures from the participants. Other outcomes measure changes in inflammation ex vivo, measuring the cytokines produced by cells taken from the participants, e.g., PBMCs upon experimental stimulation.

#### Memory

The memory aspect of Trained Immunity is important for understanding the duration of the effect of an intervention upon the innate immune system. The trials included in Table 4 vary considerably in the timescales of their measurements, with some examining changes in the innate immune system after a matter of days, e.g., NCT03332225, versus several months, e.g., NCT06257212.

Therefore, there are precedents in clinical trials in examining Trained Immunity by interrogating different aspects of this phenomenon which relate to the drug development domains highlighted in this review. These examples show some of the possibilities for clinical trial measurements but also highlight questions for trial design. Which cells should a trial examine – immune cells broadly or specific subsets? Where should Trained Immunity responses be measured, in vivo or ex vivo? What timescales should measurements take place over, days, months, or even years? Which of the many inflammatory mediators should be measured? What epigenetic changes are the most salient? These questions may be difficult to answer comprehensively ahead of conducting a clinical trial, but focusing on these questions when conducting preclinical research may grant future clinical trials the best chance of success.

## Regulatory aspects

Drug development needs to adhere to national and international rules and guidelines such as those of the International Conference of Harmonization (FDA, 2022). Further, Regulatory Agencies also develop class- or indication-specific guidelines when new areas of therapy evolve, such as the guideline for safety assessment of cancer immunotherapeutic drugs, including monoclonal antibodies, anticancer vaccines, and cytokines (Khameneh et al., 2024). These guidelines will also have to be applied to Trained Immunity-inducing drugs; however, specific guidelines for drugs targeting the innate immune system and Trained Immunity are not yet available, reflecting the novelty of this approach.

Drugs that modulate Trained Immunity fall into several therapeutic classes. The best-known approved agent that directly induces Trained Immunity is the live-attenuated bacterial vaccine BCG, authorized for tuberculosis prevention and for the treatment of non-muscle invasive bladder cancer, where its efficacy is linked to its capacity to induce Trained Immunity (Netea et al., 2020a). In Europe, another approved prescription immunomodulator is the bacterial lysate OM-85 (Broncho-Vaxom) (Röring et al., 2024), which has also been shown to act through Trained Immunity mechanisms. Furthermore, the measles, mumps, and rubella vaccine (MMR) has demonstrated similar effects (Lee et al., 2022). In addition, licensed vaccines contain adjuvants capable of stimulating Trained Immunity, such as monophosphoryl lipid A (MPLA, a TLR4 agonist), CpG oligodeoxynucleotides (TLR9 ligands), and β-glucans (dectin-1 ligands) (Lee et al., 2022). Furthermore, several small molecules and polysaccharides are in development, such as NOD2 agonists and β-glucans (Table 2).

The recognition of their impact on Trained Immunity of these prophylactic training approaches has largely come from retrospective studies. The primary intent for the development was primary vaccine-focused.

The development of modulators of Trained Immunity, whether inducers or suppressors, targeting prophylactic or therapeutic training approaches, presents distinct scientific and regulatory challenges. Regulatory Agencies are expected to require detailed elucidation of mechanisms of action and safety, extending beyond conventional concerns of off-target effects. Key open questions include whether these agents act on peripheral versus central Trained Immunity, whether they carry risks of epigenetic inheritance, and how individual immune status or microbiome profiles influence therapeutic outcomes.

Regulatory scrutiny will focus on the risks of hyperactivation or suppression of innate immunity. Critical determinants such as dose, timing, and route of administration will shape the magnitude of immune training, necessitating careful characterization of immune responses, vigilant safety monitoring, and robust pharmacovigilance. Additional uncertainties remain regarding the duration and reversibility of trained immune effects. Will the immune response to a second administration profoundly intensify the immune reaction? Will subsequent or existing infections of immune inflammatory status have an impact on the immune training?

In summary, Trained Immunity-targeting drugs must meet the highest standards of safety and efficacy. Emerging risk-based regulatory frameworks in the USA and Europe aim to address their unique immunological properties while enabling accelerated development of innovative immune-based therapies.

## RoadMap to DDD in Trained Immunity

DDD in Trained Immunity is a relatively new endeavor and therefore does not yet have many precedents or regulatory guidelines. This raises potential risks (unprecedented development pathway) but also several benefits (relatively limited competition providing room for innovation). However, Trained Immunity is involved in many different indications, and many of these are likely to have a precedented regulatory pathway to approval which can be consulted and modified to form a RoadMap for assets targeting Trained Immunity. Early interactions with Regulatory Agencies are recommended as with any other newly evolving therapeutic approach.

We have categorized the five domains which are relevant for drug development in Trained Immunity, as this facilitates the production of a RoadMap for DDD. The drug development domains categorize the mechanisms of Trained Immunity into five ‘buckets’ – epigenetic, metabolic, differentiation, inflammatory, and memory – which form a common thread linking the stages in the DDD process. These domains are used to consider targets, models, biomarkers, pharmacology, translational science, indications, and clinical trials that could be important in the RoadMap to discover and develop new medicines in the field of Trained Immunity. They form a common thread which can be traced throughout the process, from target identification, through translational science and into clinical trials.

**Figure 10.**
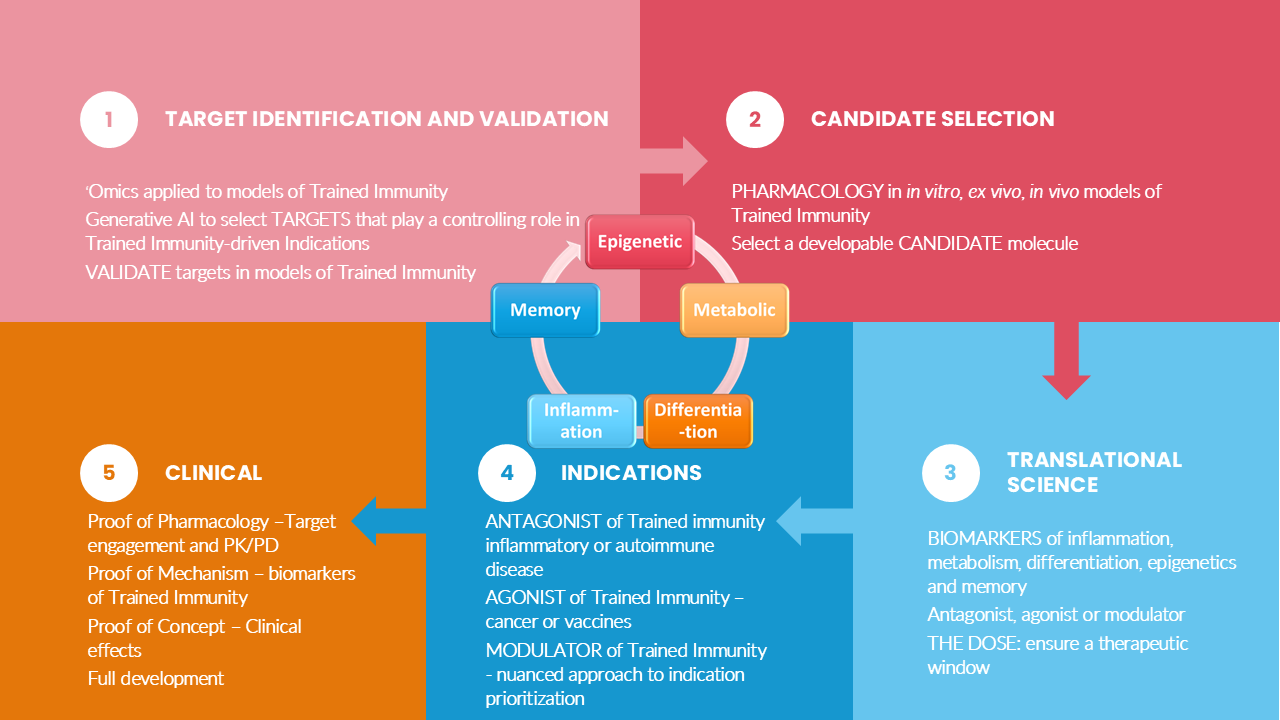
RoadMap to discovery and development of molecules that are designed to provide clinical benefit in indications relevant to Trained Immunity.

A RoadMap has been provided in this review to guide the development of new therapeutics that modulate the Trained Immunity response to provide clinical benefit for patients (Figure 10). Five major steps are described, including target identification and validation, candidate selection, translational science, indication prioritization, and clinical development. Within each of these steps is a large variety of options to achieve the planned objectives while leaving ample opportunity for innovation and originality.

It is worth noting that some areas of DDD are outside the scope of this review. These include:

- Chemistry, manufacturing, and controls (CMC): Chemistry, Manufacturing and Controls: Regulatory Considerations Through Clinical Development
- Intellectual property (IP) strategy.
- Regulatory strategy including Clinical Trial Applications: Regulatory Strategy: An Overview
- Statisticians in the Pharmaceutical Industry (PSI): PSI Homepage
- Quality, toxicology, and clinical guidelines from the International Council for Harmonisation of Technical Requirements for Pharmaceuticals for Human Use (ICH, https://www.ich.org/) and from Regulatory Agencies such as from the Food and Drug Administration (FDA).

## Appendix 1

### Detailed description of the key stages of DDD

- **Target discovery and validation:** A potential drug target, usually a protein, e.g., a receptor or an enzyme associated with a disease is discovered and is then validated to understand the potential role of the target in pathophysiology. Target validation is a critical step in the drug discovery process that aims to confirm the relevance and potential therapeutic benefits of modulating a specific molecular target. It may include in vitro assays to measure biological activity, such as expression profiling or cell-based models. These could be co-culture models of human cells to evaluate the target's behavior in a disease-relevant context.

- **Drug screening and design:** Potential drug candidates that modulate the chosen target are screened and designed using methods like high-throughput screening (HTS), fragment-based drug design, and computational chemistry. HTS involves large libraries containing millions of drug-like compounds with molecular weights around 500 Da. Fragment-based drug discovery involves screening low-molecular-weight compounds, which initially bind weakly to target proteins; however, they offer potential for further modification into potent drugs through medicinal chemistry effort. Other sophisticated methods involving rational design, which may include elements of AI-assisted design, are also being used.
- **Hit to lead phase:** Promising compounds (hits) exhibiting the desired biological activity against a target protein are evaluated and optimized to generate lead compounds. Hits are evaluated for chemical properties such as solubility, metabolic stability, bioavailability, and selectivity. This step helps rule out compounds with undesirable characteristics. Analog synthesis is performed to explore structural variations of the hits. This process aims to improve binding affinity, selectivity, and other drug-like properties.
- **Lead optimization:** Lead compounds are further refined. This includes modifying functional groups or the molecular backbone of the lead to enhance potency, selectivity, and stability. One of the most vital tools in the lead optimization process is the structure-activity relationship analysis. By systematically altering parts of a lead molecule, the effects of these changes on biological activity can be assessed. This culminates in the selection of the ‘best’ molecule, termed the candidate, to proceed into the clinical phase.
- **The preclinical phase** occurs after candidate selection but before clinical trials and involves the production of drug substance of sufficient quality and quantity to supply all the activities up to and including the early clinical trials. Once suitable material is available, PK studies in vivo may be conducted to explore the bioavailability, the plasma levels, the distribution, metabolism, and elimination of the candidate over time. PK is therefore the study of what the body does to the drug. Toxicology studies can then commence (dose-range finding, GLP toxicology in two species, genotoxicity studies, safety pharmacology). The GLP toxicology studies must be designed to provide ‘cover’ for the planned FIH trial. This means that a ‘no adverse effect level’ or in oncology the highest non-severely toxic dose must be identified in toxicology studies, and this determines the maximum dose and exposure that can be achieved in the FIH trial. Furthermore, the duration of the FIH trial is limited by the duration of the GLP toxicology studies (a 7-day GLP toxicology study means that the FIH study is allowed to proceed for a maximum of 7 days). In parallel with these activities, translational experiments will be carried out to identify biomarkers of target engagement, PD, biomarkers of mechanism, and surrogates of clinical benefit. PD is the study of what the drug does to the body. Importantly, these experiments will be used to estimate the possible therapeutic dose and whether or not a therapeutic window exists (i.e. the estimated therapeutic dose needs to be less than the maximum dose and exposure indicated by the toxicology studies). It is important to understand the preclinical ADME of the molecule in order to predict the human PK which is required for dose estimations.
- **Phase 1** refers to the early phase of clinical trials. The FIH trial is generally (other than oncology) conducted in healthy subjects. The primary objective is to understand the safety and tolerability of a single administration of escalating doses of the compound. In addition, assays will be conducted to demonstrate PK, target engagement, and PD at the site of pathology. After this single ascending dose escalation, additional healthy subjects (or in some cases patients – Phase 1b) will receive multiple administrations of escalating doses of the compound. In parallel, it may be prudent to conduct further longer-term toxicology studies so that the duration of dosing can be extended in subsequent trials.
- **Phase 2** trials consist of the clinical proof-of-concept trial (Phase 2a) where a suitable group of patients is administered an estimated therapeutic dose, and the primary objective is to demonstrate clinical efficacy. This is followed by the Phase 2b dose-ranging trial, where patients are administered up to three doses of compound (a minimally efficacious dose, an efficacious dose, and a supra-therapeutic dose). This trial allows the choice of the dose to be tested in Phase 3. Further toxicology studies are likely to be required, such as reprotoxicology, pediatric toxicology, and carcinogenicity studies. Some of these may be conducted in parallel with the Phase 3 trials.
- **Phase 3** trials are the pivotal trials that confirm the safety, tolerability, and efficacy of the compound and are used to apply for approval by the regulatory bodies. Two confirmatory trials are often required for registration. Generally, a comparator molecule is included to confirm the drugs’ effectiveness and to support reimbursement.
- **Regulatory strategy:** There are many regulatory guidelines available covering all the aspects from discovery to nonclinical and clinical requirements which need to be consulted to obtain an understanding of regulators’ expectations for a new drug candidate. Throughout the drug development phase, pre-IND/IND meetings with FDA or advice meetings with other Health Agencies, such as the EMA, can help to streamline drug development. It is a strategic imperative that can significantly improve the likelihood of successful drug registration and market entry. For novel approaches such as in the field of Trained Immunity, early interactions with health authorities are highly recommended. It is of value to, early on, think about a Target Product Profile document, which summarizes the intended use, target population, dosage, efficacy, safety, product quality, and commercial considerations, ensuring a structured approach to drug development and regulatory interactions.

### Route of administration

The route of administration and application scheme for Trained Immunity-inducing compounds can significantly impact the quality and type of immune response elicited.

Intravenous (IV) administration of BCG has been shown to be more effective in inducing Trained Immunity compared to intradermal or subcutaneous routes. IV administration allows BCG to more easily access the bone marrow, which is crucial for inducing systemic Trained Immunity effects (Sui and Berzofsky, 2024). Other routes of administration of compounds that regulate Trained Immunity include mucosal (Minute et al., 2025), topical administration of creams or hydrogels with active compounds, i.e., imiquimod (ter Steeg et al., 2021; Naik et al., 2017), intravesical instillations of BCG (van Puffelen et al., 2023), and oral administration of β-glucan (Horneck Johnston et al., 2024).

### A non-exhaustive table of potentially useful cell lines that are commercially available for the development of in vitro models of Trained Immunity

**Appendix 1—table 1.** Sources of cells that are potentially suitable for use in models of Trained Immunity.

Cells	Origin	Species	Product	Source
	**MONOCYTES**			
**Stem cells**	DYS0100 Induced; Pluripotent Stem Cell	Human	ATCC Product Code: ATCC-ACS-7030	https://www.lgcstandards.com/GB/en/search?text=monocyte%20cell%20lines
**Cell line**	J774A.1; Monocyte-macrophage	Mouse	ATCC Product Code: ATCC-TIB-67	https://www.lgcstandards.com/GB/en/search?text=monocyte%20cell%20lines
**Primary**	Peripheral Blood CD14+ Monocytes (PBMC)	Human	ATCC Product Code: ATCC-PCS-800–010	https://www.lgcstandards.com/GB/en/search?text=monocyte%20cell%20lines
**Cell line**	THP-1; Acute Monocytic Leukemia	Human	ATCC Product Code: ATCC-TIB-202	https://www.lgcstandards.com/GB/en/search?text=monocyte%20cell%20lines
**Cell line**	RAW 264.7 gamma NO (-); Macrophage	Mouse	ATCC Product Code: ATCC-CRL-2278	https://www.lgcstandards.com/GB/en/search?text=monocyte%20cell%20lines
**Cell line**	U-937; Histiocytic Lymphoma	Human	ATCC Product Code: ATCC-CRL-1593.2	https://www.lgcstandards.com/GB/en/search?text=u937
	**NATURAL KILLER CELLS**			
**Cell line**	NK-92; Natural Killer Cell	Human	ATCC Product Code: ATCC-CRL-2407	https://www.lgcstandards.com/GB/en/search?text=natural%20killer%20cells
**Cell line**	NK101	Human	RRID:CVCL_WN95	https://www.cellosaurus.org/CVCL_WN95
**Primary**	CD56+ Natural Killer Cells	Human	ATCC Product Code: ATCC-PCS-800-019	https://www.lgcstandards.com/GB/en/search?text=natural%20killer%20cells
	**MAST CELLS**			
**Primary**	CD34+ blood cells	Human		Rådinger et al., 2010
**Cell line**	LAD2	Human	ABM Cat. No.: T8157	https://www.abmgood.com/Human-Mast-Cell-Line-LAD2-t8157.html
**Cell line**	HMC1	Human	ABM Cat. No.: T8389	https://www.abmgood.com/catalog/products/56515/type/cells
**Cell line**	LUVA	Human	ABM Cat. No.: T0843	https://www.abmgood.com/immortalized-human-mast-cells-luva.html
**Cell line**	ROSA KIT WT	Human	RRID:CVCL_5G49	https://www.cellosaurus.org/CVCL_5G49
**Cell line**	ROSA KIT D816V	Human	RRID:CVCL_5G50	https://www.cellosaurus.org/CVCL_5G50
	**BASOPHILS**			
**Cell line**	KU812	Human	ATCC	https://www.atcc.org/search#q=mast%20cell&sort=relevancy&numberOfResults=24
**Bone marrow**	Immortalized	Human	ABM Cat. No.: T0527	https://www.abmgood.com/immortalized-human-bone-marrow-basophils.html
	**NEUTROPHILS**			
**Cell line**	HL-60	Human	ABM Cat. No.: T8970	https://www.abmgood.com/hl-60-cells.html
**Cell line**	PLB-985	Human	RRID:CVCL_2162	https://www.cellosaurus.org/CVCL_2162
**Cell line**	NB4	Human	RRID:CVCL_0005	https://www.cellosaurus.org/CVCL_0005
**Cell line**	Kasumi-1	Human	RRID:CVCL_0589	https://www.cellosaurus.org/CVCL_0589
**Primary**	iPSC	Human	ACS-3002	https://www.atcc.org/products/acs-3002
	**DENDRITIC CELLS**			
**Cell line**	BDCM	Human	RRID:CVCL_4613	https://www.cellosaurus.org/CVCL_4613
**Cell line**	CAL-1	Human	RRID:CVCL_5G46	https://www.cellosaurus.org/CVCL_5G46
**Cell line**	CS-1	Human	RRID:CVCL_T022	https://www.cellosaurus.org/CVCL_T022
**Cell line**	FDC-1	Human	RRID:CVCL_0F06	https://www.cellosaurus.org/CVCL_0F06
**Cell line**	MUTZ-3	Human	RRID:CVCL_1433	https://www.cellosaurus.org/CVCL_1433
	**EOSINOPHILS**			
**Cell line**	AML14	Human	RRID:CVCL_8286	https://www.cellosaurus.org/CVCL_8286
**Cell line**	EOL-1	Human	RRID:CVCL_0258	https://www.cellosaurus.org/CVCL_0258
**iPSC**	HPS1472	Human	RRID:CVCL_UN83	https://www.cellosaurus.org/CVCL_UN83
	**NONIMMUNE CELLS**			
**Cell lines**	Fibroblasts		20,869 Cell lines	https://www.cellosaurus.org/search?query=fibroblast Liu et al., 2016
	Epithelial cells		5444 Cell lines	https://www.cellosaurus.org/search?query=epithelial Naik et al., 2017

The cell lines listed in this table are examples only and form a subset of all of the available cell lines.
